# Biased IL-2 signals induce Foxp3-rich pulmonary lymphoid structures and facilitate long-term lung allograft acceptance in mice

**DOI:** 10.1038/s41467-023-36924-z

**Published:** 2023-03-13

**Authors:** Yoshito Yamada, Tuan Thanh Nguyen, Daniela Impellizzieri, Katsutaka Mineura, Rintaro Shibuya, Alvaro Gomariz, Martina Haberecker, Jakob Nilsson, César Nombela-Arrieta, Wolfgang Jungraithmayr, Onur Boyman

**Affiliations:** 1 Department of Thoracic Surgery, University Hospital Zurich, Zurich, Switzerland; 2 Department of Thoracic Surgery, Kyoto University Graduate School of Medicine, Kyoto, Japan; 3 Department of General Thoracic Surgery, Chiba University Graduate School of Medicine, Chiba, Japan; 4 Department of Immunology, University Hospital Zurich, Zurich, Switzerland; 5 Department of Dermatology, Kyoto University Graduate School of Medicine, Kyoto, Japan; 6 Department of Medical Oncology and Hematology, University Hospital Zurich, Zurich, Switzerland; 7 Computer Vision Laboratory, ETH Zurich, Zurich, Switzerland; 8 Institute of Pathology and Molecular Pathology, University Hospital Zurich, Zurich, Switzerland; 9 Faculty of Medicine, University of Zurich, Zurich, Switzerland; 10 Department of Thoracic Surgery, Medical Center – University of Freiburg, Faculty of Medicine, University of Freiburg, Freiburg, Germany

**Keywords:** Interleukins, Allotransplantation, Regulatory T cells, Lymphoid tissues, Lymphocyte differentiation

## Abstract

Transplantation of solid organs can be life-saving in patients with end-stage organ failure, however, graft rejection remains a major challenge. In this study, by pre-conditioning with interleukin-2 (IL-2)/anti-IL-2 antibody complex treatment biased toward IL-2 receptor α, we achieved acceptance of fully mismatched orthotopic lung allografts that remained morphologically and functionally intact for more than 90 days in immunocompetent mice. These allografts are tolerated by the actions of forkhead box p3 (Foxp3)^+^ regulatory T (Treg) cells that home to the lung allografts. Although counts of circulating Treg cells rapidly return to baseline following cessation of IL-2 treatment, Foxp3^+^ Treg cells persist in peribronchial and peribronchiolar areas of the grafted lungs, forming organized clusters reminiscent of inducible tertiary lymphoid structures (iTLS). These iTLS in lung allografts are made of Foxp3^+^ Treg cells, conventional T cells, and B cells, as evidenced by using microscopy-based distribution and neighborhood analyses. *Foxp3*-transgenic mice with inducible and selective deletion of Foxp3^+^ cells are unable to form iTLS in lung allografts, and these mice acutely reject lung allografts. Collectively, we report that short-term, high-intensity and biased IL-2 pre-conditioning facilitates acceptance of vascularized and ventilated lung allografts without the need of immunosuppression, by inducing Foxp3-controlled iTLS formation within allografts.

## Abstract (web summary)

IL-2/anti-IL-2 antibody complexes have been shown to facilitate the process of graft acceptance in transplantation. Here the authors use a mouse allograft model to show that IL-2 complexes promote graft acceptance and formation of inducible lymphoid structures containing Treg cells in transplanted lungs.

## Introduction

Owing to surgical and therapeutic advances, particularly the use of immunosuppressive drugs, solid organ transplantation (SOT) has become the treatment of choice for patients with end-stage organ failure^1^. However, most solid organ allografts suffer from rejection by allograft-specific T and B cells despite the continued use of immunosuppressive treatments^2,3^. Moreover, immunosuppressive drugs cause increased risk of infection, carcinogenesis, and direct damage of airway integrity^4,5^. Thus, finding a fine balance between limiting the incidence and severity of allograft rejection while minimizing immunosuppressive therapy is crucial. An alternative strategy to immunosuppressive drugs consists of approaches aimed at achieving immunological tolerance to the allograft.

Successful examples of operational immunological tolerance have so far been observed only in settings of prolonged mixed donor chimerism using combined transplantation of a human leukocyte antigen (HLA)-matched kidney with hematopoietic stem cells obtained from the same donor^1,6–9^. This approach, however, is hampered by several difficulties, including the need of an HLA-compatible living donor, a lymphoreductive pre-conditioning regimen causing immunosuppression and cytotoxicity in recipients, and a small risk of developing graft-versus-host disease.

To overcome these shortcomings, several groups have focused on the use of in vitro expanded regulatory T (Treg) cells to achieve operational tolerance toward solid organ allografts without the need of concomitant hematopoietic stem cell transplantation. Several studies are currently ongoing to investigate the safety and efficacy of autologous, in vitro expanded Treg cells in SOT^10–13^. However, multiple hurdles hamper the adoption of this approach for routine use in SOT. These include identification of the best-suited Treg cell subset for in vitro stimulation as well as counts and purity of Treg cells necessary for adoptive transfer to obtain sufficient numbers of tolerogenic Treg cells in the recipient.

An alternative option consists in the expansion of Treg cells in vivo. One such approach, already tested in mouse models of pancreatic islet cell transplantation and skin allografts, pertains to the use of interleukin-2 (IL-2)/anti-IL-2 monoclonal antibody (mAb) complexes (briefly, IL-2cx)^14–18^. A version of IL-2cx, termed CD25-biased (or CD25-directed) IL-2cx and made by mouse IL-2 complexed to anti-mouse IL-2 mAb clone JES6-1A12 (IL-2/JES6-1cx), was able to vigorously stimulate and expand CD25^high^ forkhead box p3 (Foxp3)^+^ Treg cells both in lymphoid and non-lymphoid organs^14,15,19,20^. Treatment of major histocompatibility complex (MHC)-mismatched C57BL/6 (B6) mice with IL-2/JES6-1cx shortly before transplantation resulted in the survival of BALB/c pancreatic islet cell allografts in about 50-80% of recipient mice^15^. However, grafting of full-thickness skin with multiple minor mismatches (i.e. from Sv129B6 mice) or fully allogeneic skin to B6 recipients resulted in acute rejection (AR) by the recipients, which could not be prevented by treatment with IL-2/JES6-1cx^16^.

There are several outstanding questions concerning these results on using CD25-biased IL-2/JES6-1cx in mouse models of SOT. Firstly, it is unclear whether bona fide forkhead box p3 (Foxp3)-dependent Treg cells are mechanistically involved in the observed tolerogenic effects following IL-2/JES6-1cx. Although previous studies showed a correlation of IL-2/JES6-1cx treatment-mediated increase in Foxp3^+^ Treg cells with allograft survival^15,16,21,22^, a mechanistic involvement of Foxp3^+^ Treg cells remains circumstantial. Secondly, the results obtained in pancreatic islet cell allograft transplantation might, at least to some extent, be explained by recovery of endogenous insulin production by the recipient’s pancreas, thus blurring our interpretation of the long-term treatment effect of IL-2/JES6-1cx^15^. Lastly, the tested allograft settings do not represent SOTs commonly used in the clinic.

Lung transplantation poses unique challenges by exposing the allograft to both endogenous antigens via the blood circulation and airborne environmental antigens, which are the main reasons for poor outcome in lung transplantation^2,23^. The respiratory system is home to a large number of lymphoid structures and different immune cells, which increases the risk of allogeneic immune responses. Together with the need for efficacious anti-microbial immunity at the respiratory mucosa, this situation makes immunosuppression a very challenging undertaking in lung transplantation^3,23,24^. Thus, successful induction of allogeneic tolerance is particularly important for pulmonary transplantation^25^. In this work, we address these above-mentioned outstanding issues and challenges by using CD25-biased IL-2cx in a clinically relevant and established model of SOT by using fully MHC-mismatched orthotopic lung allografting in mice^26^. We show that CD25-biased IL-2cx facilitate the long-term acceptance of vascularized and ventilated lung allografts, without the need of immunosuppression, by inducing Foxp3-controlled formation of inducible tertiary lymphoid structures (iTLS) within the allografts.

## Results

### IL-2/JES6-1cx treatment prevents acute rejection of lung allografts

**Fig. 1:**
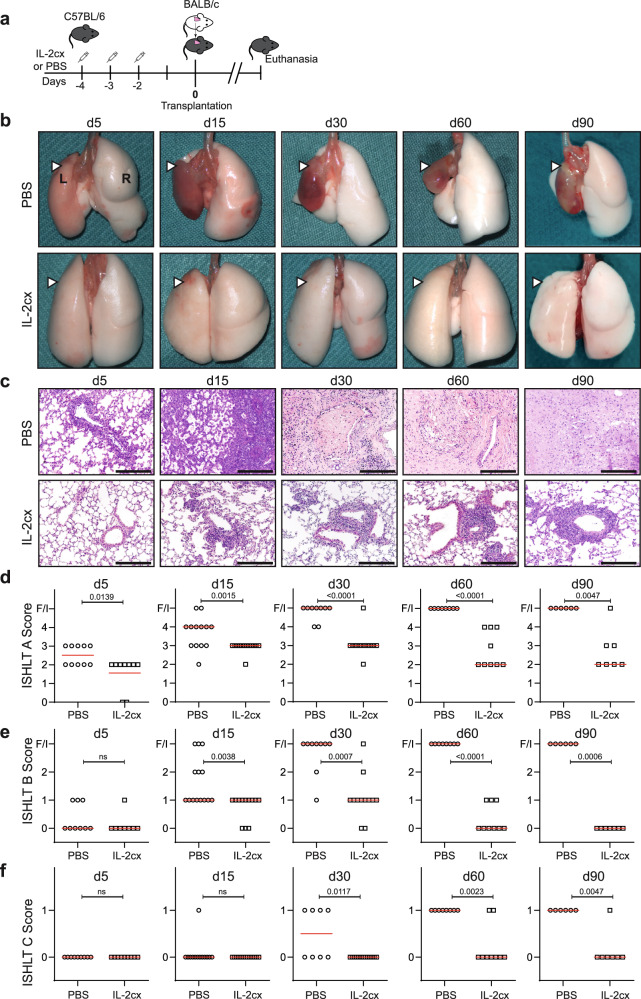
IL-2 complexes prevent acute rejection of lung allografts. **a** C57BL/6 (H-2^b^) mice were pre-treated with PBS or IL-2/JES6-1 complexes (IL-2cx) on days −4, −3, and −2, followed on day 0 by orthotopic transplantation of a left lung from BALB/c (H-2^d^) mice. **b** Macroscopic view of grafted (L, left; white arrowheads) and endogenous (R, right) lungs in PBS- (top panels) and IL-2cx-treated mice (bottom panels) on indicated days (d5–d90) after lung transplantation. **c** Representative hematoxylin and eosin (HE) staining of grafted lungs of PBS- (top panels) and IL-2cx-treated mice (bottom panels) on indicated days. Scale bars = 200 μm. **d**–**f** Scoring of rejection based on adapted International Society for Heart and Lung Transplantation (ISHLT) grade A (**d**; acute rejection), grade B (**e**; airway inflammation), and grade C (**f**; chronic airway rejection) in PBS- (open circles) and IL-2cx-treated mice (open squares). Symbols represent individual mice and horizontal red bars the median of *n* ≥ 6 mice. F/I indicates fibrotic or infarcted tissue. Data are presented as mean ± SD of *n* ≥ 6 mice of 2–3 independent experiments. Statistical comparisons in (**d**–**f**) were calculated using two-sided Mann–Whitney *U* test with calculated *p* values shown. ns not significant.

To study AR, we used a previously established mouse model of fully MHC-mismatched orthotopic lung transplantation where the left lung of a B6 mouse (H-2^b^) is replaced by a left lung of a BALB/c mouse (H-2d)^26–28^. Prior to the surgical procedure, recipients were treated on days −4, −3 and −2 with either IL-2/JES6-1cx (referred to as IL-2cx from here on) or phosphate-buffered saline (PBS) (Fig. 1a). Recipients were euthanized on days 5, 15, 30, 60, and 90 to analyze the grafted (left) and non-grafted endogenous (right) lungs macroscopically, histologically, and by flow cytometry. Compared to mice receiving PBS where AR was evident macroscopically and histologically as early as day 5 after transplantation, allograft acceptance was markedly improved in IL-2cx-treated mice (Fig. 1b). This discrepancy between the two groups became even more evident at later timepoints where lung allografts of PBS-treated animals appeared shrunken macroscopically and firm on palpation, whereas lung allografts of IL-2cx-treated recipients retained a macroscopic appearance comparable to that of the contralateral endogenous lungs for more than 90 days (Fig. 1b).

Histological analysis of lung allografts of PBS-treated mice showed signs of severe AR, according to an adapted International Society for Heart and Lung Transplantation (ISHLT) grade A scoring. This consisted of abundant leukocyte accumulations in lung alveolar septa already on day 5 (ISHLT grade A2–A3, *P* = 0.0139), which significantly worsened on subsequent timepoints (*P* = 0.0015, *P* < 0.0001, *P* < 0.0001, and *P* = 0.0047 for days 15, 30, 60, and 90, respectively) (Fig. 1c, d). Thus, leukocytes infiltrated interstitial and air-filled spaces of allografted lungs, which resulted in maximal AR scores (ISHLT grade A4; Fig. 1c, d). In contrast, grafted lungs of IL-2cx-treated recipients showed overall a normal alveolar structure and organized leukocyte accumulations starting on day 15 (Fig. 1c). Unlike the situation in PBS-treated animals, the immune cell formations in IL-2cx-treated recipients appeared to form ordered clusters and did not infiltrate the lung parenchyma (Fig. 1c). Thus, in IL-2cx-treated animals, leukocyte accumulation was preferentially localized to peribronchial, peribronchiolar and perivascular sites with only rare infiltration into alveolar septa or interstitial and air-filled spaces. ISHLT grade B scoring (airway inflammation) demonstrated that grafted lungs of PBS-treated mice reached the highest score or developed fibrotic tissues as early as 15 days after transplantation, whereas no or only mild bronchitis was observed in IL-2cx-treated animals at all timepoints investigated, including 90 days after transplantation (*P* = 0.0006; Fig. 1e). Moreover, allografted lungs of PBS-treated mice showed signs of chronic rejection on ISHLT grade C scoring (chronic airway rejection), starting from day 30 on and affecting all animals of this group on days 60 and 90 (Fig. 1f). In contrast, there was no histologic evidence of chronic rejection in the majority of IL-2cx-treated recipients up to day 90 after transplantation (*P* = 0.0047; Fig. 1f).

**Fig. 2:**
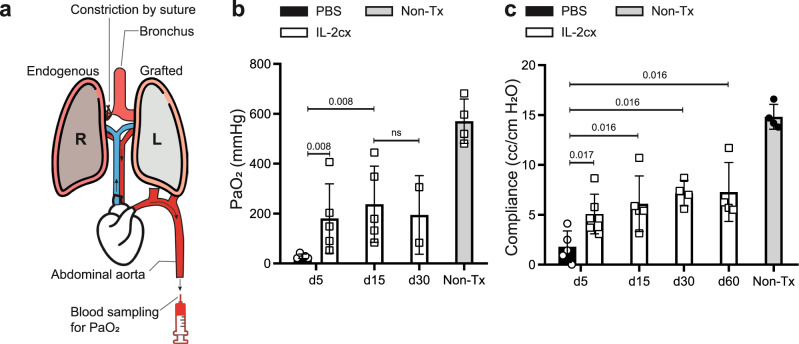
IL-2 complexes preserve lung allograft function. BALB/c lungs were transplanted into C57BL/6 recipient mice pre-treated as in Fig. 1a. **a** Scheme of experimental procedure for measurement of partial pressure of arterial oxygen (PaO_2_) and lung compliance. The endogenous lung (R) was ligated at the main bronchus by a surgical suture to prevent ventilation while the grafted lung (L) was ventilated by intubation with an intratracheal catheter. Blood was sampled from the abdominal aorta. **b**, **c** Bars represent PaO_2_ (**b**) and lung compliance (**c**; cc/cm H_2_O) of grafted lungs in PBS- (black filled bars) and IL-2cx-treated mice (open bars) on indicated days after transplantation. Non-transplanted (Non-Tx) mice served as controls. No PaO_2_ and compliance data could be obtained from PBS-treated mice from day 15 and later due to advanced rejection of grafted lungs. Data are presented as mean ± SD of *n* ≥ 6 mice of 2–3 independent experiments. Statistical comparisons in (**b**, **c**) were calculated using two-sided Mann–Whitney *U* test with calculated *p* values shown. ns not significant.

To assess vitality and functionality of lung allografts, we measured their gas exchange capacity. To this end, we ligated the right (i.e. the non-grafted) pulmonary hilum, thus leaving only the transplanted left lung ventilated, followed by blood sampling from the abdominal aorta to measure the partial pressure of oxygen (PaO_2_; Fig. 2a). This procedure was very challenging for the mice, with animals succumbing during the procedure before enough blood was obtained. For this reason, we could only obtain results for a selected number of animals and timepoints. As early as 5 days after lung transplantation, PaO_2_ levels of PBS-treated animals were significantly compromised compared to their IL-2cx-treated counterparts (*P* = 0.008; Fig. 2b). Conversely, the gas diffusion capacity of allografted lungs of IL-2cx-treated mice remained intact up to day 30 after transplantation, albeit at a lower level than in non-transplanted control animals (Fig. 2b). We observed similar results when calculating the compliance of allografted lungs based on lung volume change and air pressure applied. Thus, compared to PBS controls, allografted lungs of IL-2cx-treated mice showed substantially higher compliance values already at day 5 (*P* = 0.017) and lung compliance remained preserved up to day 60 after transplantation, although at a lower level compared to non-transplanted wild-type controls (Fig. 2c). Together, these results demonstrated that IL-2cx treatment maintained the structure and function of allografted lungs for up to 2–3 months after transplantation.

### Allograft-homing Foxp3^+^ T cells persist for extended periods

**Fig. 3:**
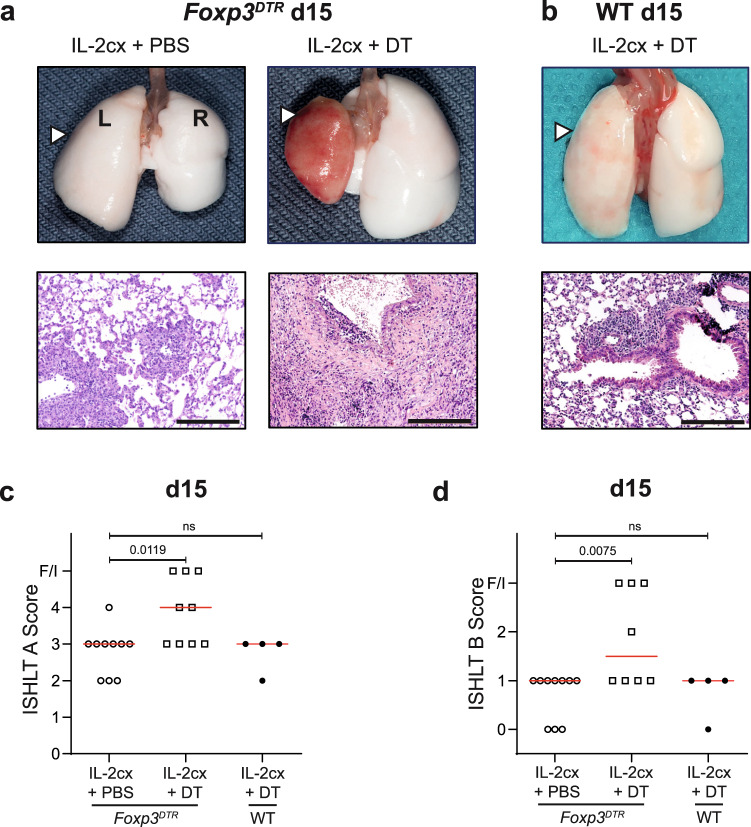
Foxp3^+^ regulatory T cells are crucial for IL-2cx-mediated lung allograft acceptance. BALB/c lungs were transplanted as in Fig. 1a into recipient mice pre-treated as indicated. **a** Macroscopic view of grafted (L; white arrowheads) and endogenous (R) lungs and representative HE stainings of grafted lungs from *Foxp3*^*DTR*^ recipient mice (on a C57BL/6 background) treated with IL-2cx + PBS (left panels) or IL-2cx + diphtheria toxin (DT; right panels). Analysis was performed on day 15 after transplantation. Scale bars = 200 μm. **b** Macroscopic view and HE staining of lungs from wild-type (WT) C57BL/6 recipient mice treated with IL-2cx + DT, and analyzed on day 15 after transplantation. Scale bar = 200 μm. **c**, **d** ISHLT grade A and grade B scores (as in Fig. 1d, e) of grafted lungs of indicated mice and treatments. Symbols represent individual mice and horizontal red bars the median of *n* = 4–10 mice. F/I indicates fibrotic or infarcted tissue. Significance of difference between groups was calculated using two-sided Mann–Whitney *U* test with calculated *p* values shown. ns not significant.

To assess whether the prolonged acceptance of lung allografts following IL-2cx treatment depended on Foxp3^+^ cells, we took advantage of *Foxp3*^*DTR*^ transgenic mice where expression of the human diphtheria toxin (DT) receptor (DTR) is driven by the Foxp3 promoter. In these mice Foxp3^+^ cells express the DTR, which allows selective depletion of Foxp3^+^ cells by administration of DT^29^. *Foxp3*^*DTR*^ mice were treated with IL-2cx as in Fig. 1a and, additionally, received either PBS or DT every second day from day 0 on. In IL-2cx-treated *Foxp3*^*DTR*^ mice receiving PBS (i.e. Foxp3^+^ Treg-proficient animals), allografted lungs showed no macroscopic signs of AR (Fig. 3a), and microscopic evaluation of allografted lungs on day 15 after transplantation (Fig. 3a) was comparable to that of IL-2cx-treated wild-type mice (Fig. 1c). Contrarily, allografts of *Foxp3*^*DTR*^ mice that received both IL-2cx and DT (the latter depleting Foxp3^+^ cells) presented clear macroscopic manifestations of AR (Fig. 3a), similar to the findings in PBS-treated wild-type animals (Fig. 1b, c). These findings were further supported by histological assessment, showing dense and disordered cellular infiltration into the allograft lung parenchyma of these mice (Fig. 3a), which was also reflected by high AR scores (Fig. 3c, d). To exclude any unspecific effects of DT, wild-type mice received lung allografts and were treated with IL-2cx and DT. Lungs of these mice remained accepted and showed a macroscopic and histological appearance and scoring (Fig. 3b–d) comparable to IL-2cx-treated wild-type mice (Fig. 1b–e).

**Fig. 4:**
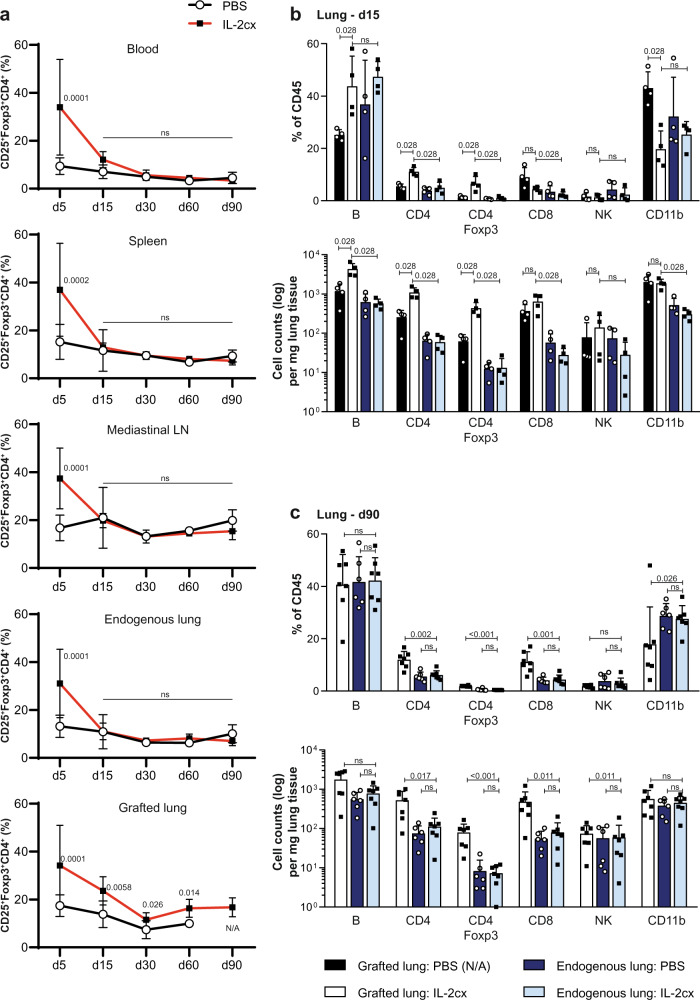
Dynamics of regulatory T cells and other immune cell subsets in different organs following treatment and lung transplantation. BALB/c lungs were transplanted as in Fig. 1a into C57BL/6 recipients pre-treated as indicated. **a** Percentages of CD25^+^ forkhead box p3 (Foxp3)^+^ CD4^+^ T cells in blood, spleen, mediastinal lymph nodes (LN), endogenous lung, and grafted lung of PBS- (open circles) and IL-2cx-treated (filled squares) lung-allografted mice on indicated days after transplantation. Data are shown as mean ± SD of *n* ≥ 4 mice of 3–7 independent experiments (exact numbers provided in Source Data file). **b**, **c** Percentages (top graphs) and total cell counts (bottom graphs) of indicated immune cell subsets in endogenous lungs and grafted lungs of PBS- and IL-2cx-treated mice, as indicated. Tissues were harvested and analyzed on days 15 (**b**) and 90 (**c**) after transplantation. No immune cells from grafted lungs of PBS-treated mice could be isolated on day 90 due to advanced fibrosis of grafted lungs (N/A, not available). Data are shown as mean ± SD of *n* ≥ 4 mice of 2–3 independent experiments. Statistical comparisons were calculated using two-sided Mann–Whitney *U* test with calculated *p* values shown. ns not significant.

Because above-mentioned data demonstrated that Foxp3-expressing cells were crucial for the establishment of lung allograft acceptance, we characterized kinetic and organ-specific changes in Foxp3^+^ cells. Foxp3 expression was almost exclusively found in CD25^+^CD4^+^ T cells, thus qualifying these cells as CD4^+^ Treg cells. On day 5 after lung transplantation, percentages of CD25^+^Foxp3^+^CD4^+^ T cells in blood, spleen, mediastinal lymph nodes (LN), endogenous lungs, and allografted lungs were significantly increased in animals receiving IL-2cx compared to PBS-treated recipients (Fig. 4a). From day 15 after lung transplantation, percentages of CD25^+^Foxp3^+^CD4^+^ T cells in IL-2cx-treated animals returned to levels seen in PBS-treated recipients in these compartments, with the exception of lung allografts in IL-2cx-treated mice where CD25^+^Foxp3^+^CD4^+^ T cell percentages remained higher than in PBS-treated controls at all timepoints investigated (Fig. 4a).

Based on these data, we performed a detailed analysis of CD25^+^Foxp3^+^CD4^+^ T cells compared to other immune cell subsets in allografted versus endogenous lungs on days 15 and 90 after transplantation. On day 15, we noticed significant increases in percentages and cell counts of B cells, CD4^+^ T cells, and CD25^+^Foxp3^+^CD4^+^ T cells in allografted lungs of IL-2cx-treated animals compared to allografted lungs of PBS-treated mice (Fig. 4b, and Supplementary Fig. 1). Counts of CD8^+^ T cells were increased on day 15 in allografted lungs of both IL-2cx- and PBS-treated recipients, compared to their endogenous lungs (Fig. 4b), indicating this reaction was likely driven by the allografts. Cell counts of CD4^+^ T cells, CD25^+^Foxp3^+^CD4^+^ T cells, and CD8^+^ T cells remained elevated in allografted lungs compared to endogenous lungs of IL-2cx-treated animals on day 90 after transplantation, whereas B cell counts returned to values seen in endogenous lungs of both IL-2cx- and PBS-treated recipients (Fig. 4c). At this timepoint, allografted lungs of all PBS-treated mice had already undergone extensive fibrosis due to rejection, which is why we could not obtain any flow cytometry data; however, the endogenous lungs of these animals were comparable to endogenous lungs of IL-2cx-treated recipients on day 90 in terms of percentages and cell counts of the aforementioned T cell subsets (Fig. 4c). Cell counts of natural killer (NK) and CD11b^+^ myeloid cells did not differ markedly on days 15 and 90 in the different groups of mice (Fig. 4b, c).

Unlike these differences in CD4^+^ T, CD25^+^Foxp3^+^CD4^+^ T, and CD8^+^ T cells in allografted lungs, percentages of CD4^+^ T, CD8^+^ T, and B cells remained unchanged over the course of observation in spleens and mediastinal LNs of IL-2cx-treated animals compared to PBS controls, except for a slight increase of CD4^+^ T cells in mediastinal LNs on day 5 (*P* = 0.033; Supplementary Fig. 2).

### Foxp3^+^ Treg cells are associated with tertiary lymphoid structures in allografts

**Fig. 5:**
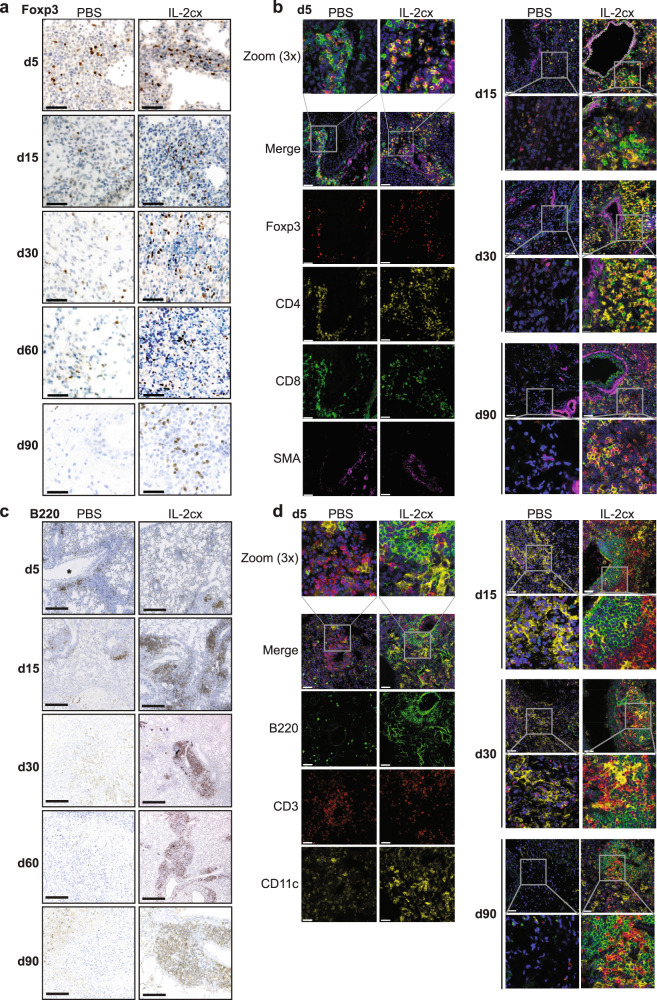
Localization of immune cell subsets in allografted lungs. BALB/c lungs were transplanted as in Fig. 1a into C57BL/6 recipients pre-treated as indicated. **a** Immunohistochemistry staining of Foxp3 in grafted lungs of PBS- (top panels) and IL-2cx-treated mice (bottom panels). Lungs were harvested on indicated days after transplantation. Scale bars = 50 μm. **b** Immunofluorescence staining for Foxp3 (red), CD4 (yellow), CD8 (green), α-smooth muscle actin (SMA; purple), and nuclei (DAPI; blue) in grafted lungs of PBS- (top panels) and IL-2cx-treated mice (bottom panels). Lungs were harvested on indicated days after transplantation. Scale bars = 50 μm. Squares indicate areas of 3X zoom. **c** Immunohistochemistry staining for B220 in grafted lungs of PBS- (top panels) and IL-2cx-treated mice (bottom panels). Lungs were harvested on indicated days after transplantation. Scale bars = 300 μm. Magnification ×25. **d** Immunofluorescence staining for B220 (green), CD3 (red), CD11c (yellow), and nuclei (DAPI; blue) in grafted lungs of PBS- (top panels) and IL-2cx-treated mice (bottom panels). Lungs were harvested on indicated days after transplantation. Scale bars = 50 μm. Squares indicate areas of 3X zoom. Representative images of *n* ≥ 4 mice of 2–3 independent experiments.

Given the durable increase of Foxp3^+^ Treg cells in lung allografts for more than 90 days, we decided to study the anatomic localization and clustering of these cells by using immunohistochemistry and fluorescence microscopy. Foxp3^+^ Treg cells were well discernible in the parenchyma of allografted lungs of both PBS- and IL-2cx-treated animals on day 5 (Fig. 5a, b, and Supplementary Fig. 3). Interestingly, between days 15 and 90 after transplantation, Foxp3^+^ Treg cells were readily found in allografted lungs of IL-2cx-treated mice, particularly in the vicinity of lung airways and blood vessels, whereas Foxp3^+^ Treg cells progressively disappeared from allografted lungs of PBS-treated animals (Fig. 5a, b, and Supplementary Fig. 3).

Similar to Foxp3^+^ Treg cells, also CD4^+^ T, CD8^+^ T, B220^+^ B, and CD11c^+^ myeloid cells gradually decreased in allografted lungs of PBS-treated recipients from day 5 to 90 after transplantation, with almost no positive staining for these cells on day 90 (Fig. 5b–d, and Supplementary Fig. 3, 4). Conversely, allografted lungs of IL-2cx-treated mice stained readily positive for CD4^+^ T, CD8^+^ T, and B cells at all timepoints assessed (Fig. 5b–d, and Supplementary Figs. 3, 4). Notably, CD4^+^ T, Foxp3^+^ Treg, CD8^+^ T, and B cells were found preferentially in the vicinity of α-smooth muscle actin (SMA)^+^ bronchi in allografted lungs of IL-2cx-treated recipients (Fig. 5b–d, and Supplementary Figs. 3, 4), suggestive of induced lymphoid clusters.

**Fig. 6:**
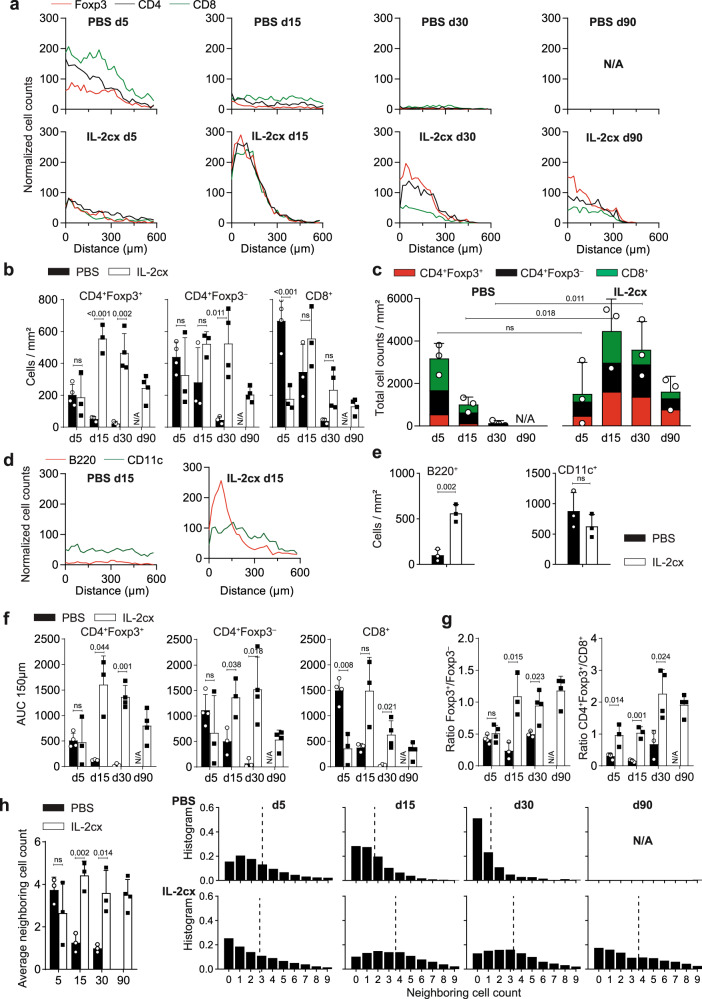
Spatial distribution of immune cell subsets in allografted lungs. BALB/c lungs were transplanted as in Fig. 1a into C57BL/6 recipients pre-treated as indicated. **a** Distance of Foxp3^+^ T (red lines), Foxp3^–^ conventional CD4^+^ T (black lines), and CD8^+^ T cells (green lines) from nearest SMA^+^ bronchus based on immunofluorescence analysis in grafted lungs of representative PBS- (top panels) and IL-2cx-treated mice (bottom panels). **b** Quantification of Foxp3^+^CD4^+^ Treg (left graph), Foxp3^–^ conventional CD4^+^ T (middle graph), and CD8^+^ T cells (right graph) based on immunofluorescence analysis of whole lungs. **c** Quantification of Foxp3^+^CD4^+^ Treg, Foxp3^–^ conventional CD4^+^, and CD8^+^ T cells in whole lungs, represented as stacked bar graphs. **d** Distance of B220^+^ B (red lines) and CD11c^+^ myeloid cells (green lines) from nearest SMA^+^ bronchus based on immunofluorescence analysis in grafted lungs of PBS- and IL-2cx-treated mice on day 15 after transplantation. **e** Quantification of B220^+^ B (left graph) and CD11c^+^ myeloid cells (right graph) based on immunofluorescence analysis. **f** Quantification of the areas under the curve (AUC) of normalized cell counts of Foxp3^+^CD4^+^ Treg, Foxp3^–^ conventional CD4^+^, and CD8^+^ T cells within 150 μm from the nearest SMA^+^ bronchus. **g** Ratios of CD4^+^Foxp3^+^ Treg over CD4^+^Foxp3^–^ T cells and of CD4^+^Foxp3^+^ Treg over CD8^+^ T cells in total lungs. **h** Quantification of cells neighboring T cells, shown in overview (left graph) and as representative examples in indicated conditions and at indicated timepoints. Data are presented as mean ± SD of *n* ≥ 3 mice of 2–3 independent experiments. Significance of difference in (**b**, **e**–**h**) was calculated using two-sided Student’s *t* test with calculated *p* values shown.

We used multiple approaches to obtain unbiased and precise information on the localization and clustering of Foxp3^+^ Treg, CD4^+^ T, and CD8^+^ T cells in lung allografts. Firstly, we annotated the positions of these cells and calculated the distance to the nearest SMA^+^ bronchus in immunofluorescence stainings of allografted lungs. Numerous CD8^+^ and CD4^+^ T cells and, to a lesser extent, Foxp3^+^ Treg cells were found in the vicinity of SMA^+^ bronchi in allografted lungs of PBS-treated animals on day 5; however, these T cell subsets and their ordered localization disappeared at day 15 and later timepoints (Fig. 6a, and Supplementary Fig. 5a). In contrast, in allografted lungs of IL-2cx-treated recipients, Foxp3^+^ Treg, CD4^+^ T, and CD8^+^ T cells were all preferentially clustered in the vicinity of SMA^+^ bronchi already on day 5, and these T cell subsets and their clustering was maintained until 90 days after transplantation (Fig. 6a, and Supplementary Figs. 5b, 6).

Moreover, quantification of Foxp3^+^ Treg, Foxp3^–^ conventional CD4^+^ T, and CD8^+^ T cells in lung allograft sections analyzed by immunofluorescence (Fig. 6b) confirmed our findings obtained by using counting beads in flow cytometry (Fig. 4b, c). Thus, in allografted lungs of PBS-treated animals, all three T cell subsets were present on day 5, and they gradually decreased on days 15 and 30 and disappeared on day 90 (Fig. 6b). In contrast, in allografted lungs of IL-2cx-treated mice, all three T cell subsets were detectable at all timepoints analyzed, with the highest cell counts per square millimeter obtained on days 15 and 30 (Fig. 6b). When comparing cell counts of PBS- and IL-2cx-treated recipients, we observed significantly higher counts of CD8^+^ T cells in lung allografts of PBS-treated animals on day 5, whereas Foxp3^+^ Treg cells were significantly higher in lung allografts of IL-2cx-treated mice on day 15 and later timepoints compared to PBS controls (Fig. 6b, c).

We also assessed distribution and counts of B220^+^ B cells and CD11c^+^ myeloid cells on day 15. B cells and myeloid cells did not show any preferential distribution in lung allografts of PBS-treated mice (Fig. 6d). Conversely, these cell subsets were preferentially localized in the vicinity of SMA^+^ bronchi in allografted lungs of IL-2cx-treated animals (Fig. 6d). Comparison of cell counts between PBS- and IL-2cx-treated recipients showed significantly more B cells in IL-2cx-treated mice, whereas counts of CD11c^+^ myeloid cells were indifferent (Fig. 6e).

Furthermore, we analyzed the areas under the curve (AUC) of normalized cell counts of immune cell subsets within 150 μm from the nearest SMA^+^ bronchus, which delimited the areas of induced lymphoid tissues around bronchi. This analysis demonstrated that the dynamic changes of T cell subsets observed in whole lungs happened in close vicinity of bronchi. Thus, in allografted lungs of PBS-treated animals, we observed numerous cells, consisting mainly of CD8^+^ T cells and, to lesser extents, also Foxp3^–^CD4^+^ T cells and Foxp3^+^ Treg cells, in AUC 150-μm areas on day 5, all of which rapidly declined at subsequent timepoints (Fig. 6f). This was also reflected by the ratios of Foxp3^+^CD4^+^ Treg over Foxp3^–^CD4^+^ T cells and of Foxp3^+^CD4^+^ Treg over CD8^+^ T cells in total lungs, which were below one on day 5 and thereafter (Fig. 6g). Conversely, allografted lungs of mice receiving IL-2cx showed similar counts of Foxp3^+^CD4^+^ Treg, Foxp3^–^CD4^+^ T, and CD8^+^ T cells in AUC 150-μm areas on day 5, with significant and sustained increases in counts of Foxp3^+^CD4^+^ Treg and Foxp3^–^CD4^+^ T cells on day 15 and later (Fig. 6f). This was also mirrored by the ratios of Foxp3^+^CD4^+^ Treg over Foxp3^–^CD4^+^ T cells and of Foxp3^+^CD4^+^ Treg over CD8^+^ T cells in total lungs, which increased to and remained at one or more from day 15 on (Fig. 6g).

These data demonstrated that in allografted lungs of IL-2cx-treated animals, Foxp3^+^CD4^+^ Treg cells as well as conventional CD4^+^ T cells and CD8^+^ T cells preferentially accumulated around bronchi and localized within lymphoid clusters, suggestive of iTLS. To estimate the interactions of Foxp3^+^CD4^+^ Treg cells with conventional CD4^+^ T cells and CD8^+^ T cells, we quantified how many neighboring cells a given T cell in each condition and timepoint had. This analysis showed that T cells had on average 3.5 neighboring cells on day 5, decreasing to below two cells on day 15 and to about one cell on day 30 in allografts of animals receiving PBS (Fig. 6h). In contrast, in allografted lungs of IL-2cx-treated recipients, the average counts of T cell neighbors was about two on day 5 and increased to about 4 on day 15 and thereafter (Fig. 6h). These changes were also mirrored at the level of T cell subsets (Supplementary Fig. 7). Furthermore, evidence of peripheral node addressin (PNAd)^+^ cells in allografted lungs of IL-2cx-treated animals was suggestive of the presence of high endothelial venule (HEV)-like blood vessels (Supplementary Fig. 8). Taken together, these data suggested that IL-2cx fostered the formation and maintenance of iTLS consisting of Foxp3^+^ Treg, conventional T, and B cells.

### Foxp3^+^ cells are essential for induction of allograft-associated iTLS

**Fig. 7:**
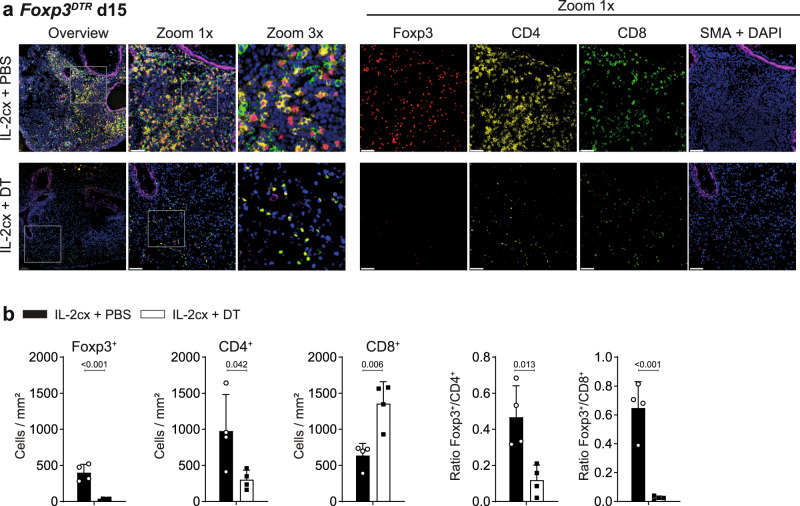
Depletion of Foxp3^+^ Treg cells disrupts ordered distribution of immune cells in allografted lungs. BALB/c lungs were transplanted as in Fig. 1a into recipient mice pre-treated as indicated. **a** Immunofluorescence staining for Foxp3 (red), CD4 (yellow), CD8 (green), SMA (purple), and nuclei (DAPI; blue) in allografted lungs from *Foxp3*^*DTR*^ mice (on a C57BL/6 background) treated with IL-2cx + PBS (top panels) or IL-2cx + DT (bottom panels). Analysis was performed on day 15 after transplantation. Squares indicate areas of zoom-ins. Scale bars = 50 μm. **b** BALB/c lungs were transplanted as in (**a**). Immunohistochemistry analysis of Foxp3, CD4, and CD8 in allografted BALB/c lungs was performed on day 15 after transplantation, followed by quantification of Foxp3^+^ Treg, CD4^+^, and CD8^+^ T cells and calculation of their ratios, as indicated. Data are presented as mean ± SD of *n* = 4 mice of 2 independent experiments. Significance of difference was calculated using two-sided Student’s *t* test with calculated *p* values shown.

To assess whether Foxp3^+^CD4^+^ Treg cells, rather than other IL-2-sensitive immune cells, were crucial for the formation of iTLS in lung allografts, we analyzed allografted lungs in IL-2cx-treated *Foxp3*^*DTR*^ mice receiving either PBS (resulting in Foxp3^+^ cell proficiency) or DT (Foxp3^+^ cell deficiency). Compared to their Foxp3^+^ Treg cell-proficient counterparts, iTLS were absent in animals deficient in Foxp3^+^ cells following administration of DT (Fig. 7a). To exclude any unspecific effects of DT, wild-type animals were given the same treatment with IL-2cx and concomitant DT. In these mice, iTLS formation was not affected by the administration of DT (Supplementary Fig. 9). Moreover, quantification of T cell subsets by immunohistochemistry indicated that counts of Foxp3^+^ Treg cells, CD4^+^ T cells, and CD8^+^ T cells were comparable in animals receiving IL-2cx plus PBS, resulting in ratios of Foxp3^+^ Treg cells to CD4^+^ or CD8^+^ T cells in favor of Treg cells, whereas mice treated with IL-2cx plus DT contained markedly altered cell counts and ratios (Fig. 7b).

## Discussion

By using short-term treatment with CD25-biased IL-2cx in a mouse model of fully MHC-mismatched orthotopic lung transplantation, we achieved allograft survival for more than 90 days without the use of maintenance immunosuppressive therapy. Mechanistically, CD25-biased IL-2cx expanded Foxp3^+^ Treg cells which persisted in lung allografts and correlated with the formation of induced lymphoid clusters around bronchi, bronchioli and blood vessels, which were reminiscent of iTLS. These iTLS were rich in Foxp3^+^ Treg cells, and also contained CD4^+^ T, CD8^+^ T, B, and CD11c^+^ myeloid cells, as well as PNAd^+^ HEV-like blood vessels. The absence of IL-2cx-mediated Foxp3^+^ Treg cell expansion, either by lack of IL-2cx or depletion of Foxp3^+^ Treg cells in *Foxp3*^*DTR*^-transgenic mice, prevented the induction of iTLS in lung allografts and allograft tolerance. Thus, these findings link IL-2cx treatment to Foxp3^+^ Treg cell-associated iTLS formation in lung allograft survival.

The formation of tertiary lymphoid structures (TLS), also called ectopic lymphoid structures, has been associated previously with effector immune responses in autoimmune disease and cancer^30–34^. TLS have been described in salivary glands of patients with active Sjörgen’s syndrome and in synovial tissues of patients with rheumatoid arthritis^30,31^. In allograft-directed immune responses, the contribution of TLS appears to be more controversial, as detailed below.

An adverse effect of TLS in transplantation was suggested based on observations in a rat chronic allograft rejection model as well as in human kidney and lung allografts undergoing chronic rejection^31,35–39^. In these situations, TLS have been proposed to function as ectopic germinal centers facilitating the survival of alloantigen-specific B cells and local production of alloantigen-specific antibodies. Conversely, other studies have argued for a beneficial function of TLS in allograft tolerance. TLS-like lymphoid infiltrates have been described in portal zones of liver allografts in patients achieving operational tolerance^40^. These infiltrates, composed primarily of Foxp3^+^CD4^+^ Treg cells and conventional CD4^+^ and CD8^+^ T cells, were shown to persist for several years. Moreover, in mouse models of kidney and lung transplantation, the formation of TLS correlated with allograft tolerance^41–45^. Hence, in a mouse lung allograft model, formation of TLS in lung allografts was associated with graft tolerance^42^, and allograft TLS-associated Foxp3^+^ Treg cells inhibited alloantigen-specific antibody production^43^. Thus, whereas some studies have associated the presence of TLS with chronic allograft rejection, others have argued for a beneficial contribution of TLS in allograft tolerance. Also in our allograft setting, we observed the formation of lymphoid structures that resembled TLS, which might be a sign of low-grade chronic rejection. However, we favor the hypothesis that the presence of iTLS in our lung allograft model represents an ongoing and active immune response, the outcome of which depends on the counts, proximity and activity of Foxp3^+^ Treg cells and effector CD4^+^ and CD8^+^ T cells. While our induction treatment using IL-2cx resulted in preferential proliferation and activation of Foxp3^+^ Treg cells during the first two weeks after transplantation, the vicinity of allograft-homing Foxp3^+^ Treg cells to effector T cells within iTLS likely provided them with prosurvival signals, such as by IL-2, ensuring their prolonged survival. We hypothesize that these Foxp3^+^ Treg cell subsets, in turn, kept alloantigen-specific effector CD4^+^ and CD8^+^ T cells in check, which resulted in the observed sustained and stable ratios of Foxp3^+^ Treg cells to effector T cells within iTLS of lung allografts of IL-2cx-treated animals. Moreover, we found these iTLS also contained PNAd^+^ blood vessels. Although we could not detect vascular cell adhesion molecule 1 on these blood vessels, we think they represented HEV-like vessels^46^.

Furthermore, our data demonstrated that Foxp3^+^ Treg cells were necessary for iTLS formation and survival of lung allografts. Thus, the depletion of Foxp3^+^ Treg cells abrogated iTLS development and allograft acceptance and maintenance. By molecularly linking IL-2cx-expanded Foxp3^+^ Treg cells to allograft survival, these findings extend previous studies investigating IL-2cx in mouse allograft models. Accordingly, Pilat and colleagues used IL-2cx in a mouse model of skin allograft rejection and observed that combination of IL-2cx with rapamycin and a neutralizing anti-IL-6 mAb resulted in long-term allograft survival^22^. However, when they concomitantly administered an anti-CD25 mAb to animals receiving the said combination treatment, the mice rejected their skin allografts, which the authors interpreted as dependency of allograft tolerance on CD25^+^Foxp3^+^ Treg cells^22^. Whereas their interpretation is probably correct, anti-CD25 mAb can also deplete other CD25^+^ regulatory immune cell subsets, including Foxp3^–^CD4^+^ Treg cells, regulatory CD8^+^ T cells, and regulatory innate lymphoid cells^47–49^. Unlike the administration of an anti-CD25 mAb, our approach using *Foxp3*^*DTR*^ mice allowed selective depletion of Foxp3^+^ cells, thus demonstrating that allograft tolerance following IL-2cx was indeed dependent on Foxp3^+^ cells.

Although our findings provide insights into the immunological processes following lung allograft transplantation and IL-2cx treatment, several questions arise. Firstly, it remains to be elucidated whether IL-2cx affected counts and functions of thymus-derived or also of peripherally induced Treg cells. Previous publications suggested that CD25-biased IL-2cx did not convert CD25^–^ conventional T cells to CD25^+^ Treg cells in the periphery, but they stimulated preexisting thymus-derived CD25^+^ Treg cells^15^. However, these experiments were done in resting mice, whereas the inflammatory environment in the allograft might produce the necessary T cell receptor signals and transforming growth factor-β, which – together with IL-2cx – might stimulate the development of peripherally induced Treg cells^50^. Moreover, CD25-biased IL-2cx could enhance the suppressive function of Treg cells by increasing their CD25, CD39, cytotoxic T lymphocyte antigen-4, and Foxp3 expression^14,15,51^. Thus, both increased counts and boosted suppressive functions of circulating and lung allograft-infiltrating Foxp3^+^ Treg cells in IL-2cx-treated recipients likely contributed to the prevention of acute allograft rejection in these mice.

Secondly, allograft-infiltrating Foxp3^+^ Treg cells accumulated around bronchi, bronchioli and blood vessels, thus forming Treg cell-rich iTLS. Whether Foxp3^+^ Treg cells induced these iTLS or were attracted by iTLS that formed without Treg cells, are interesting questions. By comparing cell counts and spatial distribution of Foxp3^+^ Treg cells, we observed that Foxp3^+^ Treg cells were as numerous as CD8^+^ T cells in lung allografts of IL-2cx-treated recipients on day 5, which was maintained until day 90, whereas in PBS-treated mice, CD8^+^ T cells were significantly higher already on day 5, which correlated with acute allograft rejection in these animals. Thus, we favor the hypothesis that Foxp3^+^ Treg cells were more functional in IL-2cx-treated mice on day 5, which enabled them to suppress the acutely activated CD8^+^ and conventional CD4^+^ T cells. The subsequent clustering of Treg cells with conventional CD4^+^ T cells, the latter known to produce IL-2^50^, in allograft iTLS of IL-2cx-treated recipients likely created a microenvironment that ensured homeostatic survival of Treg cells^52^; this latter mechanism might explain the comparable proportions of cell counts of graft-infiltrating Treg to conventional CD4^+^ T cells in these animals. Moreover, it is conceivable that IL-2 signals also contributed to the activation and/or expansion of other immune cell subsets in allograft iTLS, including regulatory B and innate lymphoid cells^53,54^. These issues should be investigated in future studies. Furthermore, it will be interesting to assess whether CD25-biased IL-2cx affect anti-microbial immunity in the respiratory tract. On this point, a recent study showed that CD25-biased IL-2cx positively affected intranasal influenza A virus infection in mice by reducing virus-induced pulmonary immunopathology and improving fitness of anti-viral CD4^+^ T cells^55^.

In combination with medical adversities associated with long-term immunosuppressive treatment, allograft dysfunction—in particular due to chronic rejection—is the major factor limiting graft survival and life expectancy of patients undergoing SOT^24,56,57^. The field of clinical SOT would benefit from strategies inducing durable tolerance toward allografts without the need of immunosuppression. Induction of long-term allograft tolerance by modulation of Treg cells—by the use of CD25-biased IL-2cx, as shown in this study, or other CD25-biased IL-2 formulations—is a promising approach to address these unmet needs.

## Methods

### Mice

3-month-old female C57BL/6J mice, mice expressing diphtheria toxin (DT) receptor under the *Foxp3* promoter (*Foxp3*^*DTR*^; B6 background; The Jackson Laboratory), and BALB/c (Charles River) mice were purchased from indicated suppliers. Mice were maintained in a specific pathogen-free facility at University Hospital Zurich at 22 °C room temperature, 40–60% humidity, on a 12-h light-dark cycle (7 a.m. to 7 p.m.), and given food and water ad libitum, according to institutional guidelines. Animal experiments received prior approval by the veterinary office of Canton of Zurich (license number ZH240/15), following pre-established exclusion criteria, and were conducted in accordance with Swiss Federal and Cantonal laws. Certain revision experiments were conducted at Kyoto University (institutional license number Med Kyo 22569) in accordance with Japanese laws.

### Lung transplantation and in vivo treatments

IL-2/anti-IL-2 mAb complexes (IL-2cx)^14^ were prepared freshly by mixing 1 μg recombinant mouse IL-2 (BioLegend, UK) and 5 μg of JES6-1A12 (BioXCell, USA) at a molar ratio of 2:1. Mice received three consecutive intraperitoneal injections on days −4, −3 and −2 before lung transplantation and were sacrificed on days 5, 15, 30, 60, and 90 after lung transplantation.

Orthotopic lung transplantation was performed as described previously^26–28^, comprising five different groups of mice (donor lung → recipient) receiving (i) phosphate buffer saline (PBS) in BALB/c → C57BL/6, (ii) IL-2cx in BALB/c → C57BL/6, (iii) IL-2cx + PBS in BALB/c → *Foxp3*^*DTR*^, (iv) IL-2cx + DT in BALB/c → *Foxp3*^*DTR*^, and (v) IL-2cx + DT in BALB/c → C57BL/6. Lungs were flushed with 2 mL 0.9% normal saline solution at a pressure of 10 cm H_2_O via the pulmonary artery, and organs were harvested. Grafted and endogenous lungs were washed with PBS and divided into two parts for flow cytometry and histological analysis.

### Flow cytometry

Single-cell suspensions of lungs, LNs, spleen, and blood were prepared following published protocols^58–61^. Cells were stained for flow cytometry analysis using PBS containing 1% FCS and 2 mM EDTA with fluorochrome-conjugated antibodies. Antibodies are listed in Supplementary Table 1. For Foxp3 staining, cells were fixed and permeabilized with a staining kit (eBioscience) according to the manufacturer’s instructions. Data were acquired using BD LSR II Fortessa and analyzed by FlowJo (both BD Biosciences).

### Microscopy

Lungs, spleen, and mediastinal LNs were fixed in 4% buffered formalin and embedded in paraffin following published protocols^58,62^. 4 μm-thick tissue sections were stained with hematoxylin and eosin or using immunohistochemistry on Bond-Max with BOND Polymer Refine Detection kit (Leica Biosystem) according to the manufacturer’s instructions. Primary antibodies used for histological analysis were anti-rabbit CD3 (IR503, DAKO), anti-mouse CD4 (14-9766, eBioscience), anti-mouse CD8 (14-0808, eBioscience), anti-rat B220 (550539, BD Pharmingen), anti-rabbit FoxP3 (ab54501, Abcam). Antibody detection required antigen retrieval with 10 mM citrate at pH 6. Secondary antibodies were anti-rat IgG/HRP (A 5795, Sigma-Aldrich), anti-goat/HRP (P0160, DAKO), and anti-rabbit/HRP (K4003, DAKO), followed by 3,3’-diaminobenzidine substrate kit (Thermo Fisher Scientific) for detection. Nuclei were stained with hematoxylin. Sections were analyzed using ImageScope for image acquisition (Aperio Technologies, Inc.).

For confocal microscopy, lungs were embedded in optimum cutting temperature compound (OCT; Tissue-Tek) and cut into 7–12 μm thick sections using a cryostat (HM550, Thermo Fisher Scientific). Sections were fixed with cold acetone and blocked with 5% BSA in PBS for 1 h at room temperature. Staining was performed using fluorochrome-conjugated antibodies overnight at 4 °C. Antibodies are listed in Supplementary Table 2. Nuclei were stained using 4′,6-diamidin-2-phenylindol (DAPI). Images were acquired with SP5 and SP8 confocal laser scanning microscopes (Leica).

### Distribution of immune cells in lungs

Immunofluorescence images were analyzed using Imaris 9.3 (Bitplane). Cells expressing B220, CD4, CD8, CD11c, and Foxp3 were manually annotated in Imaris, while SMA-positive bronchi and a mask delineating the tissue were manually annotated using Microscopy Image Browser 2.6 (ref. ^63^). Distribution of different cell subsets around bronchi was assessed, as previously published^64^. Briefly, distances to the closest pixel of a bronchus were calculated for each of the pixels within annotated tissues (empty space distance; ESD) and for each cell type. These distances were represented as a cumulative distribution function (CDF) for assessing their position to the ESD, which served as reference. Similarly, we performed a cell neighborhood analysis by quantifying cell counts of total cells and of individual T cell subsets in the 20-μm vicinity of a given T cell subset in indicated conditions and at indicated timepoints.

### Assessment of rejection score

The rejection scores were evaluated according to an adaptation of the standard criteria by the International Society for Heart and Lung Transplantation (ISHLT)^65^. Grade A (acute rejection) was analyzed in a blinded fashion by three independent investigators, including a certified pathologist. Grade B (airway inflammation: bronchitis, bronchiolitis) and grade C (chronic airway rejection: obliterative bronchiolitis) were analyzed by a certified pathologist. Examples for scoring are shown in Supplementary Fig. 10. Tissues showing advanced rejection and fibrosis or infarction were designated as “F/I”.

### Lung functionality and compliance

For measurement of partial pressure of arterial oxygen (PaO_2_) and lung compliance, the endogenous lung was constricted at the level of the main bronchus by a surgical suture to prevent ventilation while the transplanted lung was ventilated by intubation with an intratracheal catheter, as published^66^. Lung compliance was measured with a VentElite small animal ventilator (Harvard Apparatus, USA). Where indicated, recipients of lung allografts were selected randomly for PaO_2_ measurement. Mice were sedated with ketamine and xylazine, ventilated with FiO_2_ 100%, and the right bronchus leading to the non-transplanted lung was ligated. Subsequently, 100 μl or more of arterial blood from the abdominal aorta were withdrawn to measure PaO_2_ levels. Only mice intended for functional testing underwent hilum ligation, and lung tissues from these mice were not further used for histological or flow cytometry analysis to avoid possible artifacts.

### Statistical analyses

The numbers of mice used in each experiment are indicated in figure legends. Results are presented as means ± standard deviation. Statistical significance was calculated as described in figure legends. For most experiments, Mann–Whitney *U* tests were used for calculation of *p* values. Statistical analysis was performed with Graph-Pad Prism 8 software. Statistical significance was established at 95% confidence level (*P* < 0.05).

### Reporting summary

Further information on research design is available in the Nature Portfolio Reporting Summary linked to this article.

## Supplementary information

Supplementary Information

Reporting Summary

## Data Availability

All relevant data generated in this study are provided in the manuscript and the Supplementary Information. Source data are provided with this paper.

## Source data

Source Data
