# Supplementary material for: Biased IL-2 signals induce Foxp3-rich pulmonary lymphoid structures and facilitate long-term lung allograft acceptance in mice
Source: Nat Commun. 2023 Mar 13;14:1383. doi: 10.1038/s41467-023-36924-z (PMC10011523; doi:10.1038/s41467-023-36924-z)
Supplement: Supplementary file 1 — Supplementary Information [file 41467_2023_36924_MOESM1_ESM.pdf]

Supplementary Information to

Biased IL-2 signals induce Foxp3-rich pulmonary lymphoid structures and facilitate long-term lung allograft acceptance in mice

Yoshito Yamada, Tuan Thanh Nguyen, Daniela Impellizzieri, Katsutaka Mineura, Rintaro Shibuya, Alvaro Gomariz, Martina Haberecker, Jakob Nilsson, César Nombela-Arrieta, Wolfgang Jungraithmayr, and Onur Boyman

This Supplementary Information file contains:

Supplementary Fig. 1. Flow cytometry gating strategies.

Supplementary Fig. 2. Dynamics of B cells and T cell subsets in secondary lymphoid organs following treatment and lung transplantation.

Supplementary Fig. 3. Localization of T cell subsets in allografted lungs.

Supplementary Fig. 4. Localization of B and CD11c^{+} cells in allografted lungs.

Supplementary Fig. 5. Spatial distribution of immune cell subsets in allografted lungs.

Supplementary Fig. 6. Cumulative distribution function of immune cell subsets.

Supplementary Fig. 7. Analysis of T cell neighbors in allografted lungs.

Supplementary Fig. 8. Peripheral node addressin staining.

Supplementary Fig. 9. Use of diphtheria toxin in IL-2cx-treated wild-type mice.

Supplementary Fig. 10. Representative histological examples illustrating scoring.

Supplementary Table 1. Antibodies used for flow cytometry.

Supplementary Table 2. Antibodies used for immunofluorescence staining.

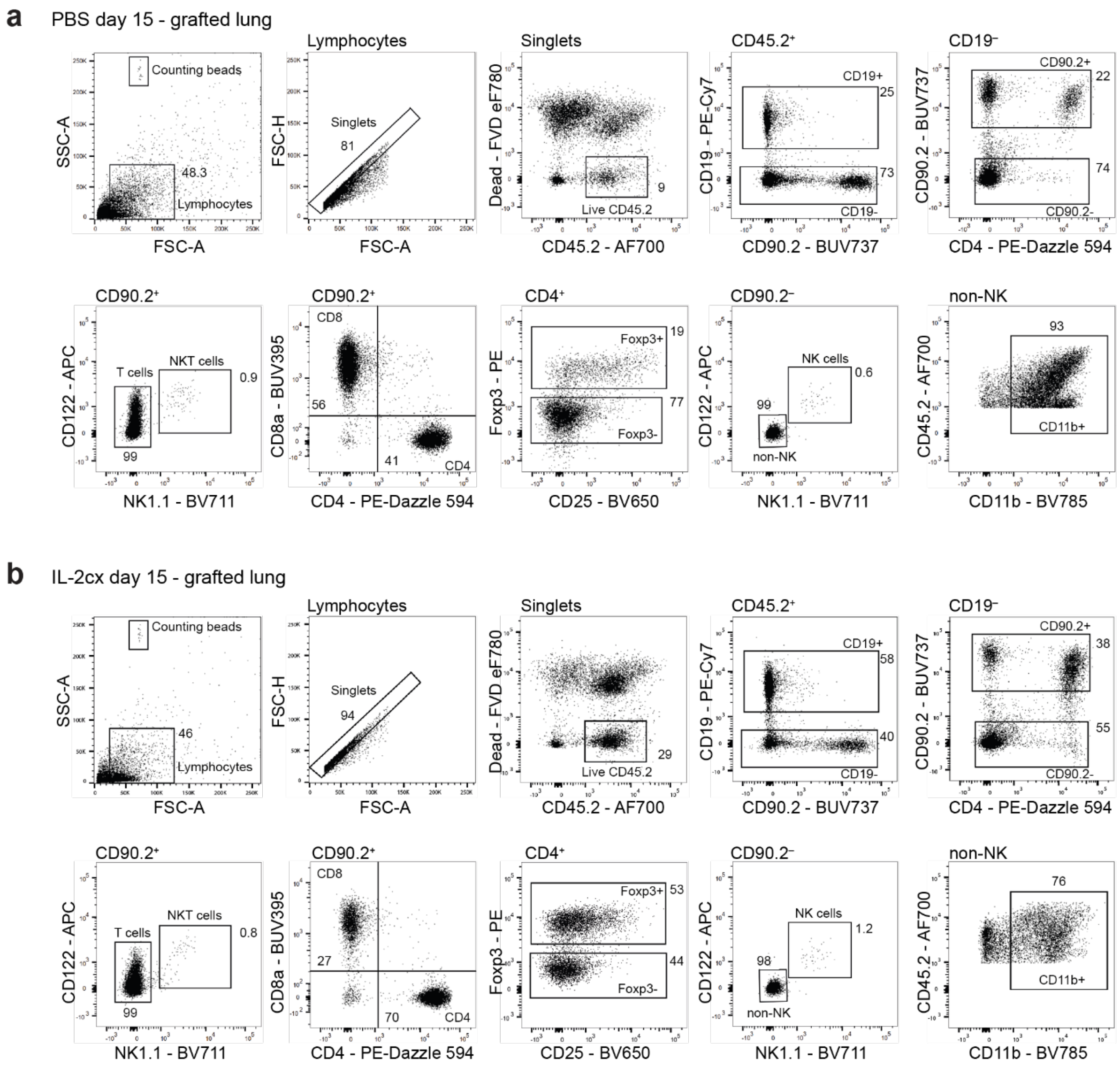

Supplementary Fig. 1. Flow cytometry gating strategies. (**a, b**) Representative gating strategy of indicated immune cell subsets in lung tissues of PBS- (**a**) and IL-2cx-treated mice (**b**), analyzed on day 15 after transplantation.

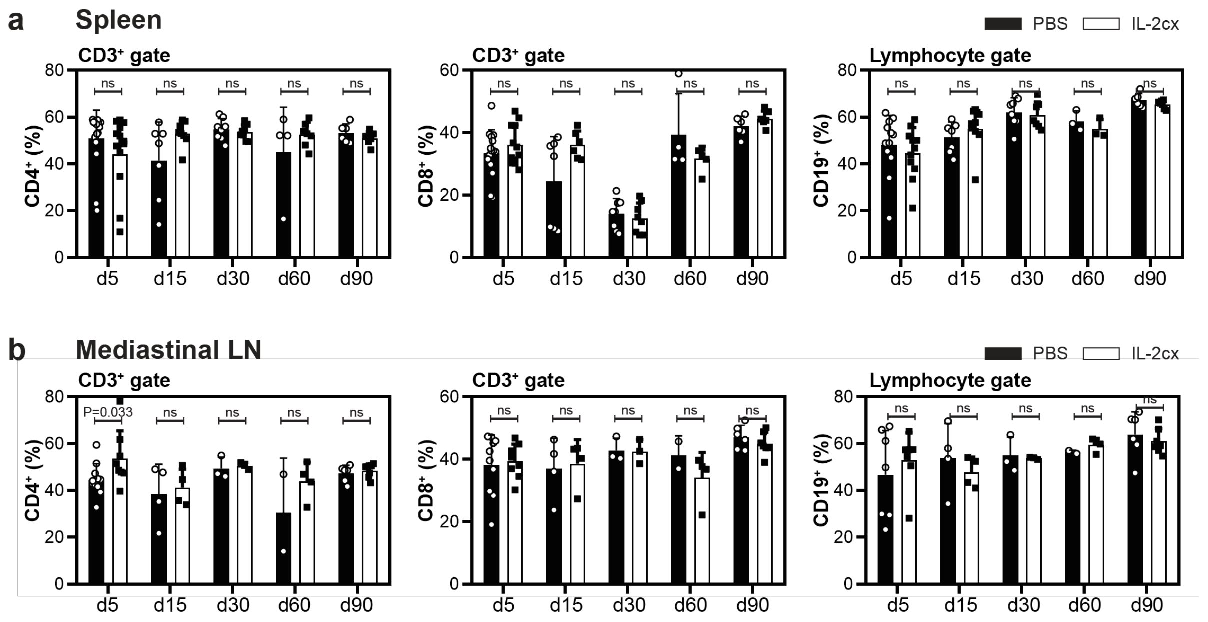

Supplementary Fig. 2. Dynamics of B cells and T cell subsets in secondary lymphoid organs following treatment and lung transplantation. (**a, b**) Bars represent percentages of indicated T cell subsets in spleen (**a**) and mediastinal lymph nodes (LN) (**b**) of PBS- (open circles and black filled bars) and IL-2cx-treated mice (filled squares and open bars). Analysis was performed on indicated days after transplantation. Each symbol represents an individual mouse. Data are shown as mean ± SD of $n \geq 4$ of 3–7 independent experiments (exact number provided in Source Data file). Significance of differences between groups was calculated using two-sided Mann-Whitney U test with calculated p values shown. ns, not significant.

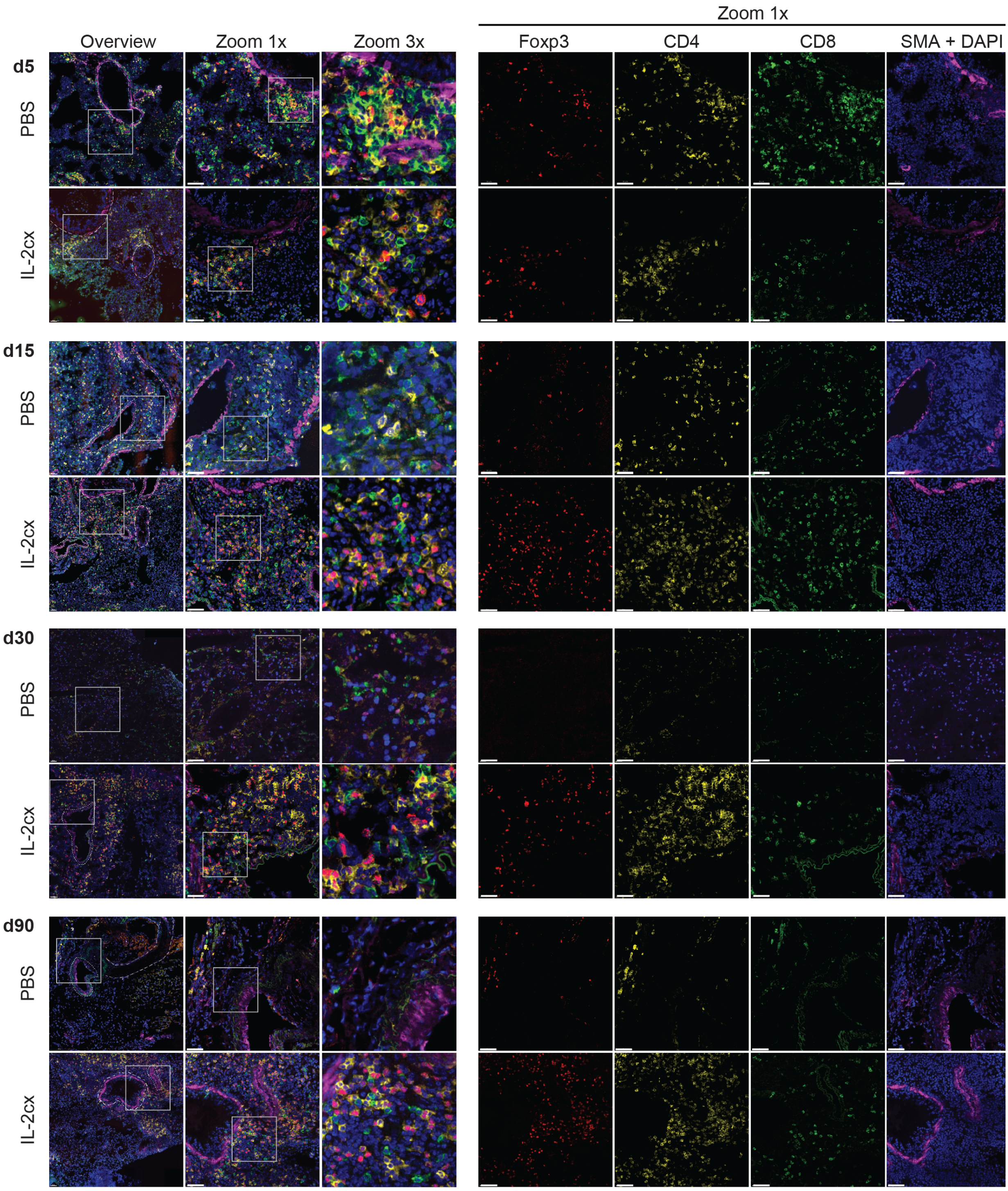

Supplementary Fig. 3. Localization of T cell subsets in allografted lungs. BALB/c lungs were transplanted as in Fig. 1a into C57BL/6 recipients pre-treated as indicated. Immunofluorescence staining for Foxp3 (red), CD4 (yellow), CD8 (green), α-smooth muscle actin (SMA; purple), and nuclei by 4′,6-diamidino-2-phenylindole (DAPI; blue) in grafted lungs of PBS- (top panels) and IL-2cx-treated mice (bottom panels). Lungs were harvested on indicated days after transplantation. Squares indicate areas of zoom-ins. Scale bars = 50 µm.

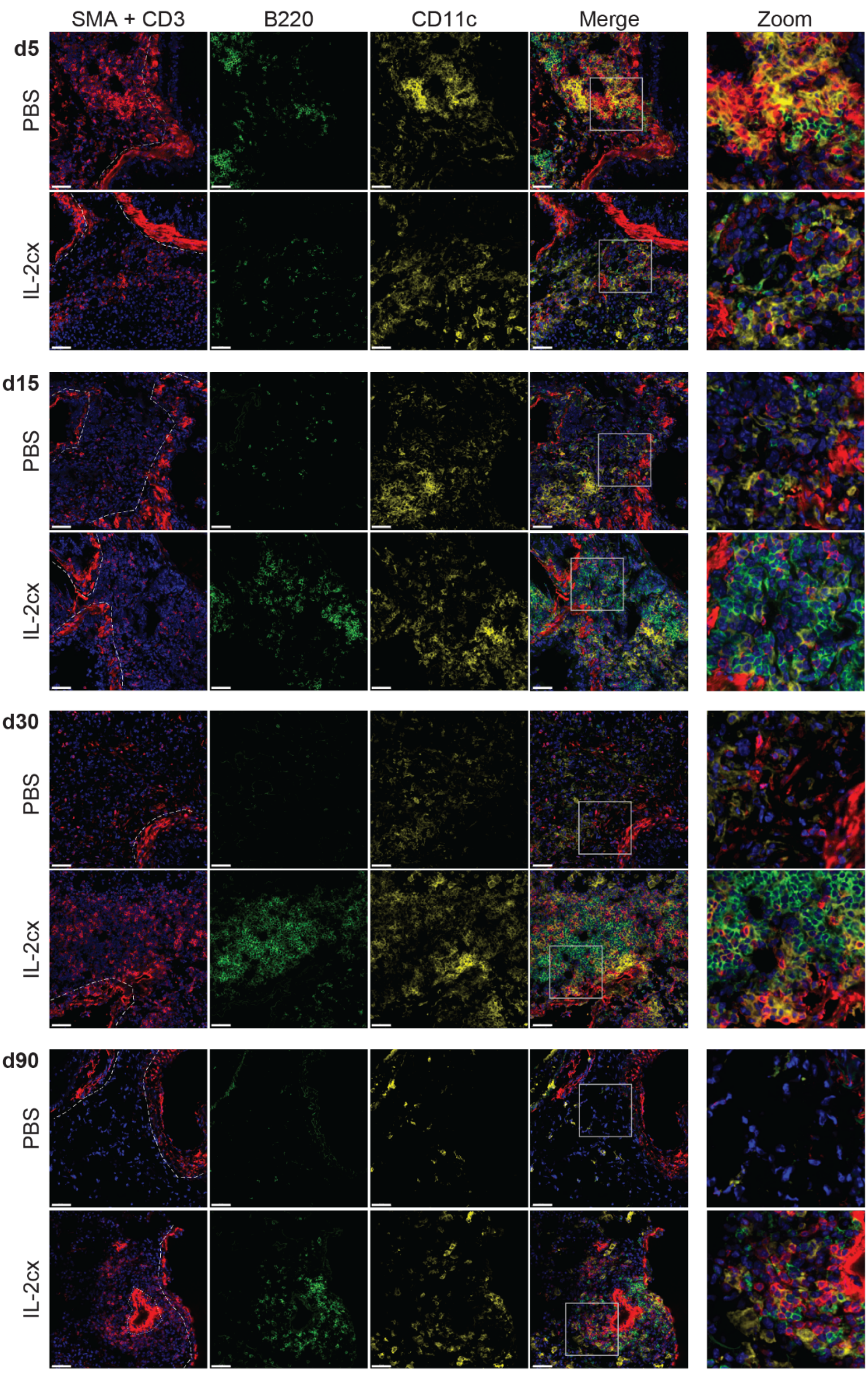

Supplementary Fig. 4. Localization of B and CD11c^{+} cells in allografted lungs. BALB/c lungs were transplanted as in Fig. 1a into C57BL/6 recipients pre-treated as indicated. Immunofluorescence staining for SMA and CD3 (red), B220 (green), CD11c (yellow), and nuclei (DAPI; blue) in grafted lungs of PBS- (top panels) and IL-2cx-treated mice (bottom panels). Lungs were harvested on indicated days after transplantation. Squares indicate areas of 3X zoom. Scale bars = 50 µm.

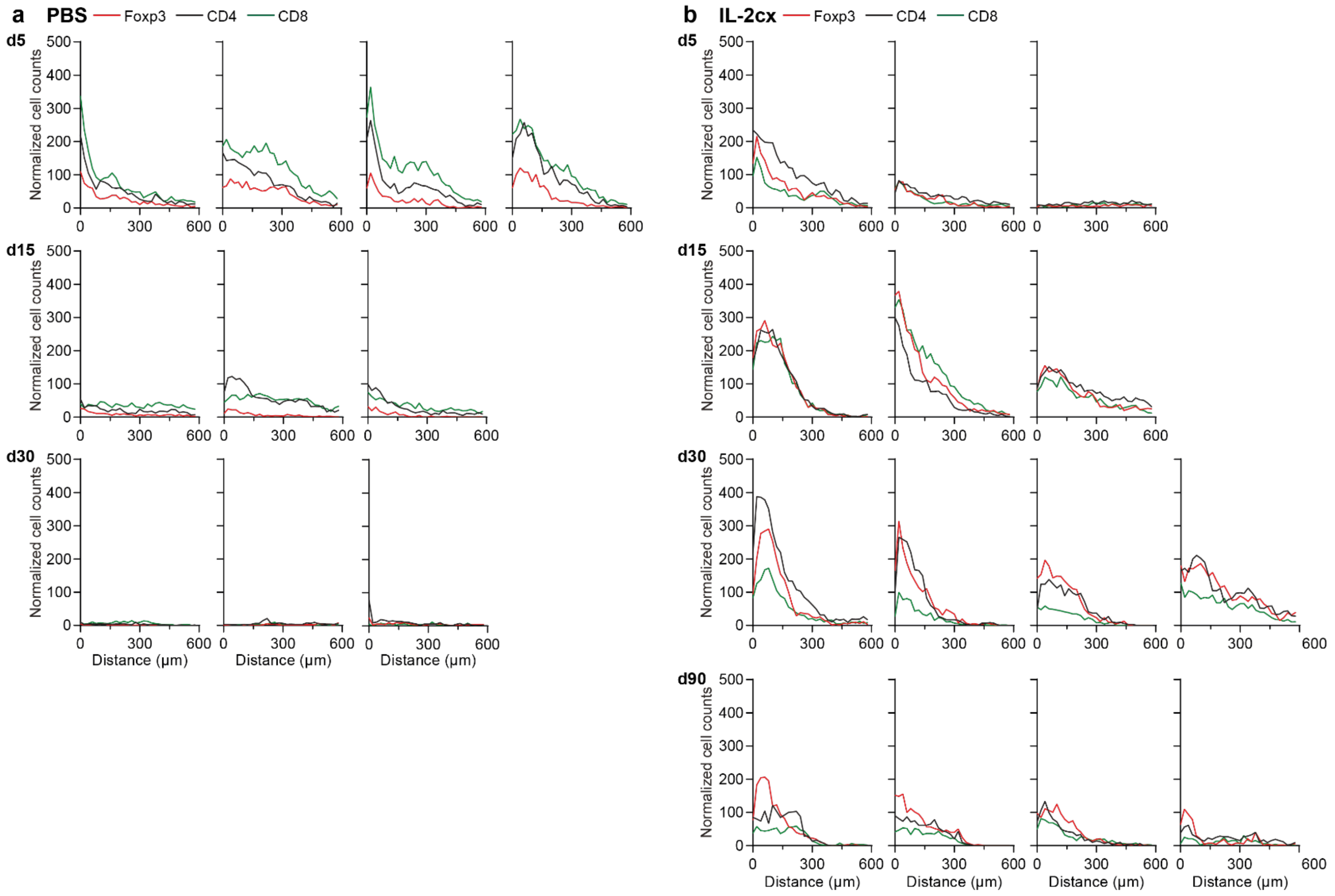

a PBS
Foxp3
CD4
CD8
d5
d15
d30
Normalized cell counts
Distance (µm)
b IL-2cx
Foxp3
CD4
CD8
d5
d15
d30
d90
Normalized cell counts
Distance (µm)

Supplementary Fig. 5. Spatial distribution of immune cell subsets in allografted lungs. BALB/c lungs were transplanted as in Fig. 1a into C57BL/6 recipients pre-treated as indicated. (**a, b**) Distance of Foxp3$^+$ T (red lines), Foxp3$^-$ conventional CD4$^+$ T (black lines), and CD8$^+$ T cells (green lines) from nearest SMA$^+$ bronchus based on immunofluorescence analysis in grafted lungs of individual PBS- (**a**) and IL-2cx-treated mice (**b**) on indicated days after transplantation.

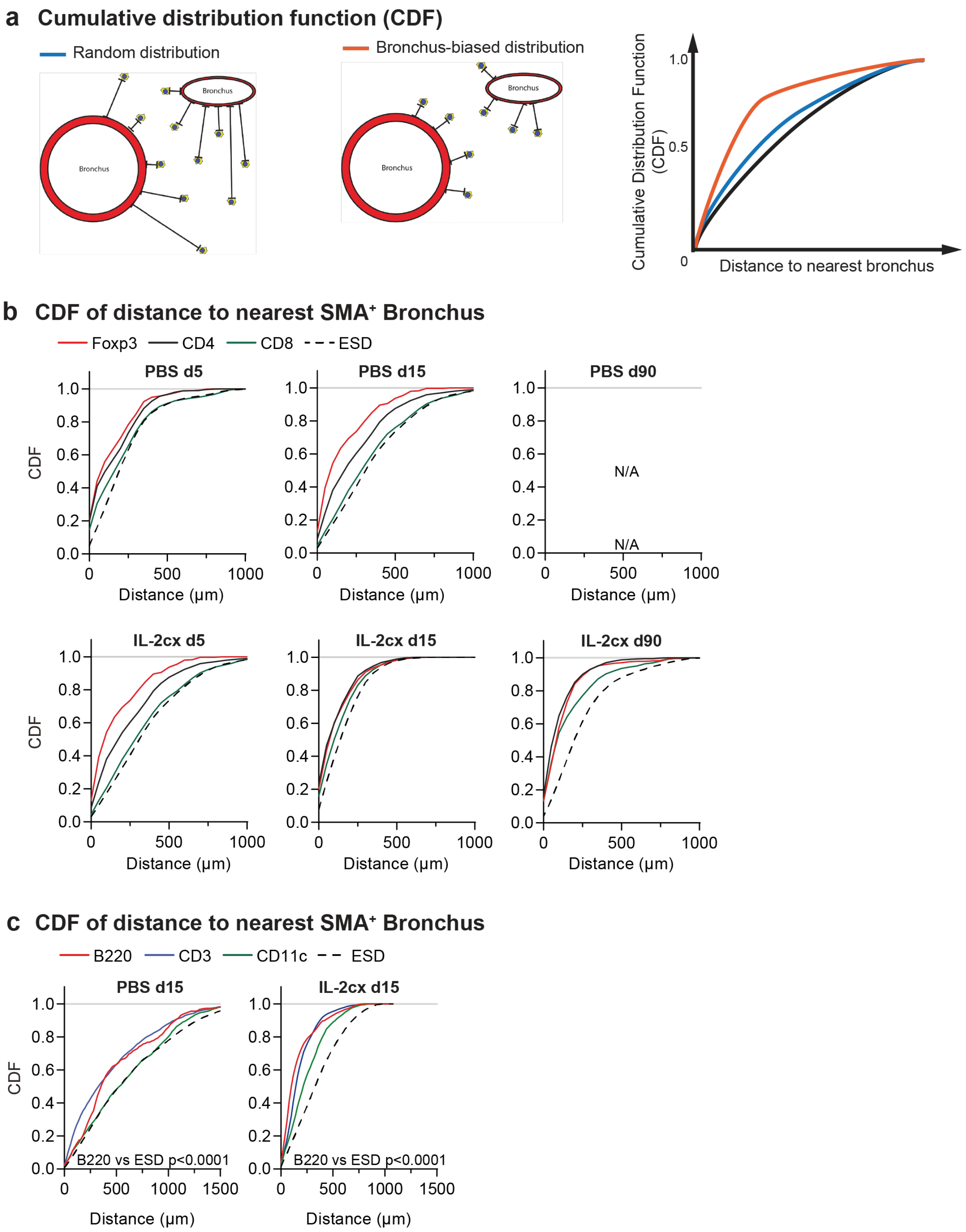
a Cumulative distribution function (CDF)
Random distribution
Bronchus-biased distribution
Bronchus
Cumulative Distribution Function (CDF)
1.0
0.5
0
Distance to nearest bronchus
b CDF of distance to nearest SMA+ Bronchus
Foxp3
CD4
CD8
ESD
PBS d5
PBS d15
PBS d90
N/A
IL-2cx d5
IL-2cx d15
IL-2cx d90
CDF
Distance (μm)
c CDF of distance to nearest SMA+ Bronchus
B220
CD3
CD11c
ESD
PBS d15
IL-2cx d15
B220 vs ESD p<0.0001
Distance (μm)

Supplementary Fig. 6. Cumulative distribution function of immune cell subsets. (**a**) Graphical representation of the cumulative distribution function (CDF) calculating the distance of indicated immune cell subsets to the nearest bronchus in allografted lungs. The two examples illustrate cells randomly distributed around a bronchus (left panel) or cells distributed in proximity to a bronchus (middle panel). These two examples of CDF are represented in the right panel by a blue line for random distribution and an orange line for bronchus-biased distribution. The black line indicates empty space distribution (ESD) and represents random cell distribution. A shift of the CDF away from ESD indicates a bronchus-biased distribution. (**b**) CDFs of $Foxp3^{+}CD4^{+}$ Treg (Foxp3; red lines), $Foxp3^{-}$ conventional $CD4^{+}$ T (CD4; black lines), and $CD8^{+}$ T cells (CD8; green lines) were calculated based on immunofluorescence staining in allografted lungs of PBS- and IL-2cx-treated mice on days 5, 15 and 90 after transplantation. (**c**) CDFs of $B220^{+}$ B (red lines), $CD3^{+}$ T (blue lines), and $CD11c^{+}$ myeloid cells (green lines) were analyzed and calculated as described in **b**.

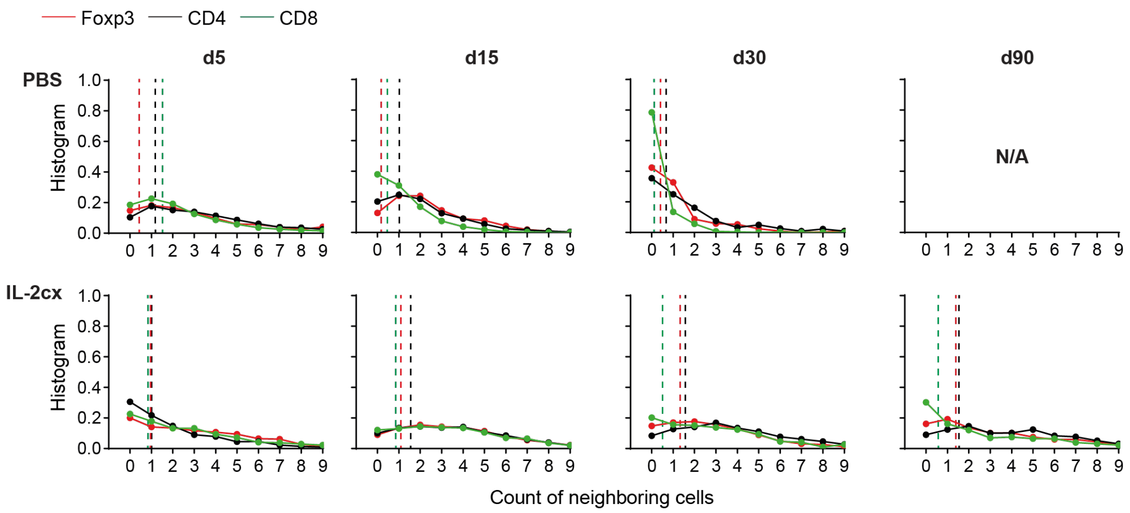

Supplementary Fig. 7. Analysis of T cell neighbors in allografted lungs. BALB/c lungs were transplanted as in Fig. 1a into C57BL/6 recipients pre-treated as indicated. Cells neighboring T cell subsets were quantified in indicated conditions and at indicated timepoints, with CD4$^+$Foxp3$^+$ Treg cells represented as red dots and lines, CD4$^+$Foxp3$^-$ T cells as black dots and lines, and CD8$^+$ T cells as green dots and lines. Dashed and colored vertical lines represent the mean counts of neighboring cells.

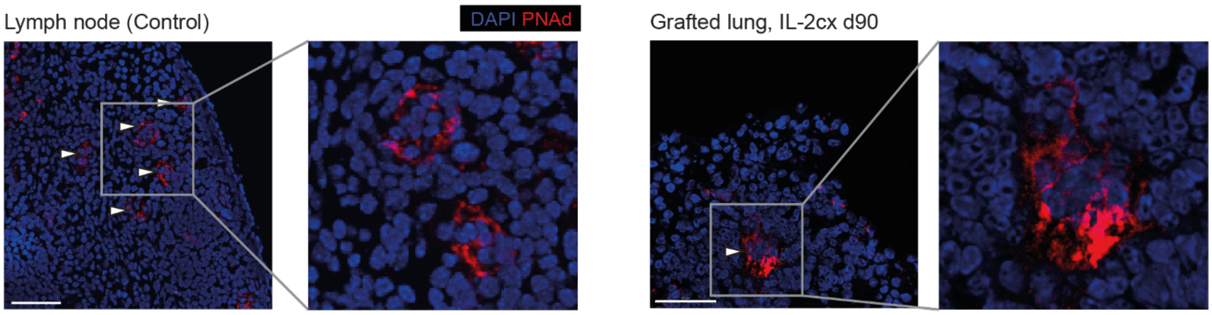

Supplementary Fig. 8. Peripheral node addressin staining. Immunofluorescence staining for peripheral node addressin (PNAd; red) and nuclei (DAPI; blue) in a lymph node of an untreated C57BL/6 WT control (left) and in a grafted lung of an IL-2cx-treated mouse (right) on day 90 after transplantation. White arrow heads indicate PNAd^{+} cells. Scale bars = 150 µm. Representative images of n = 3 mice of 2 independent experiments.

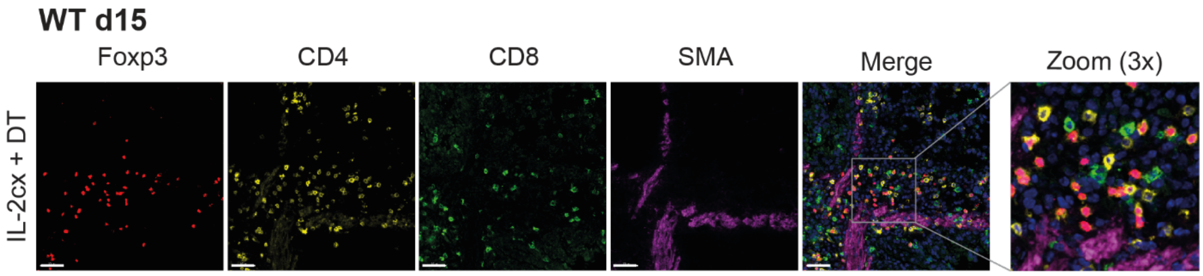

Supplementary Fig. 9. Use of diphtheria toxin in IL-2cx-treated wild-type mice. BALB/c lungs were transplanted as in Fig. 1a into IL-2cx-treated C57BL/6 wild-type recipient mice, which additionally received diphtheria toxin (DT) every second day. Shown are immunofluorescence staining for Foxp3 (red), CD4 (yellow), CD8 (green), SMA (purple), and nuclei (DAPI; blue) in allografted lungs from C57BL/6 wild-type mice treated with IL-2cx + DT. Analysis was performed on day 15 after transplantation. Square indicates area of 3X zoom. Scale bars = 50 µm. Representative images of n = 3 mice of 2 independent experiments.

a **Exemplary histology samples for ISHLT A score**

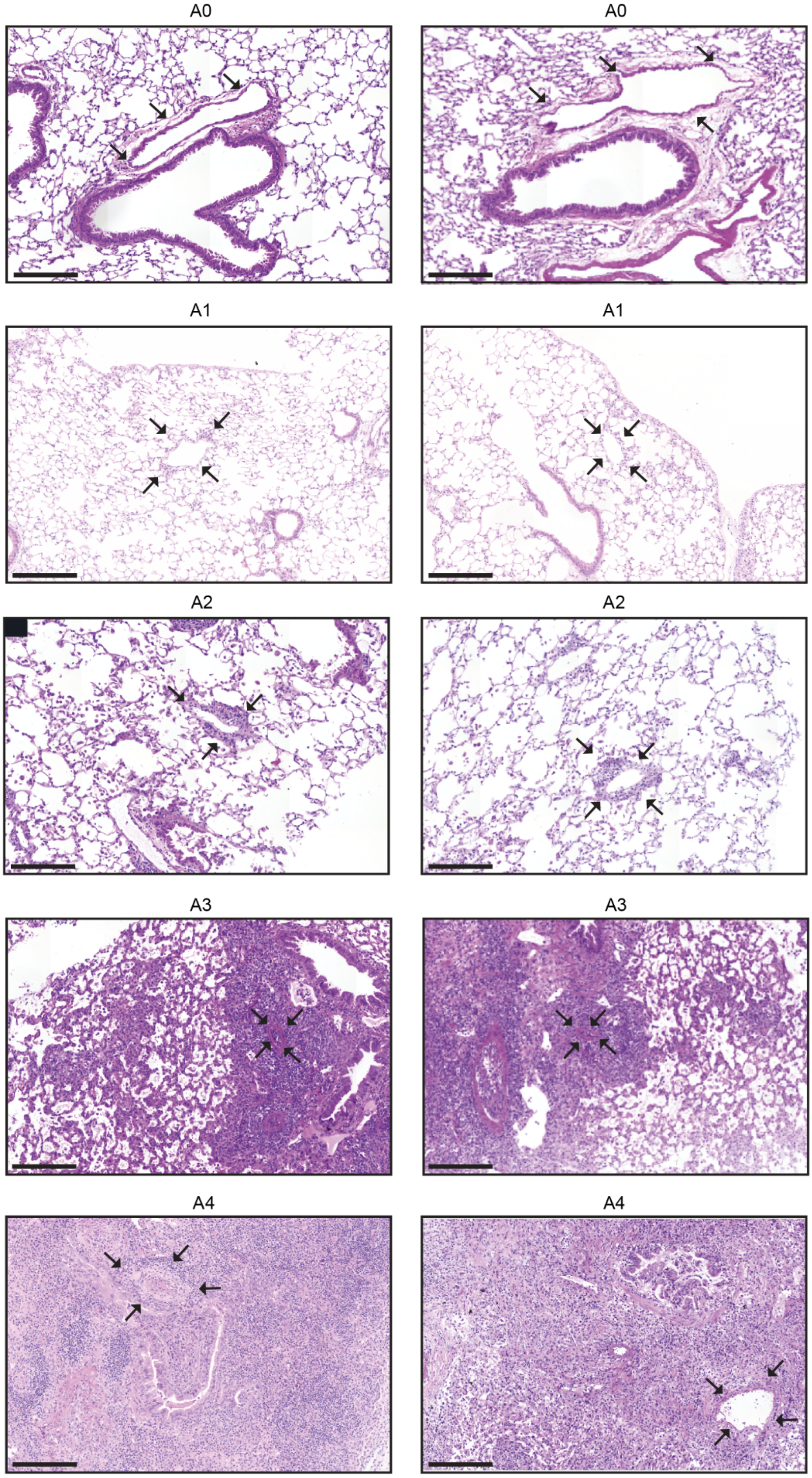

b **Exemplary histology samples for ISHLT B score**

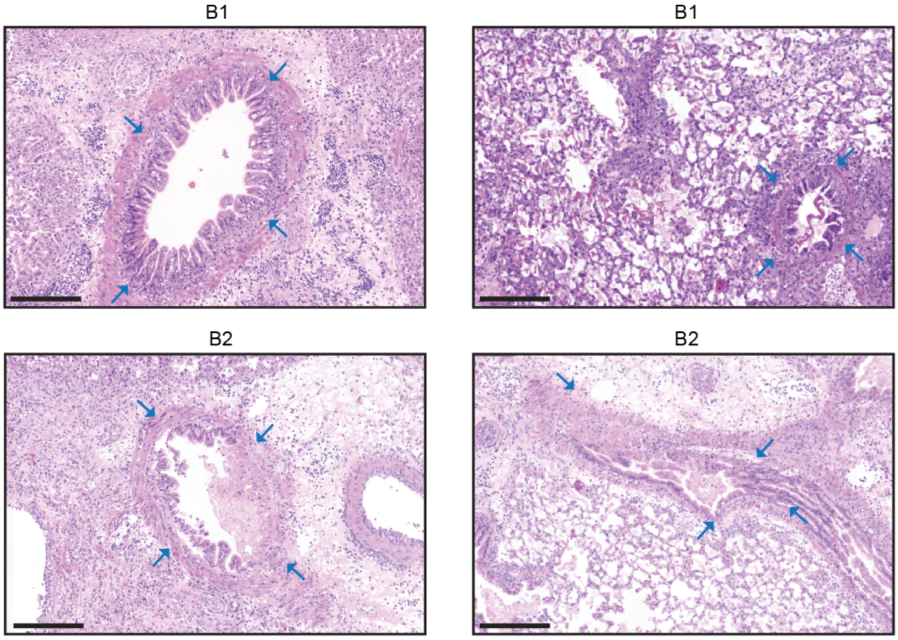

c **Exemplary histology samples for ISHLT C score**

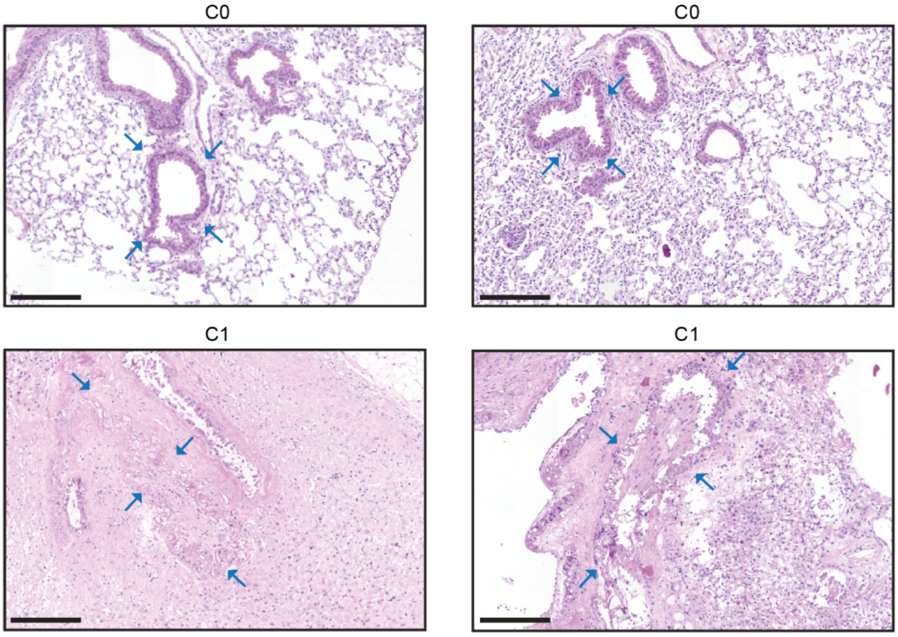

d **Exemplary histology samples for fibrosis and infarction**

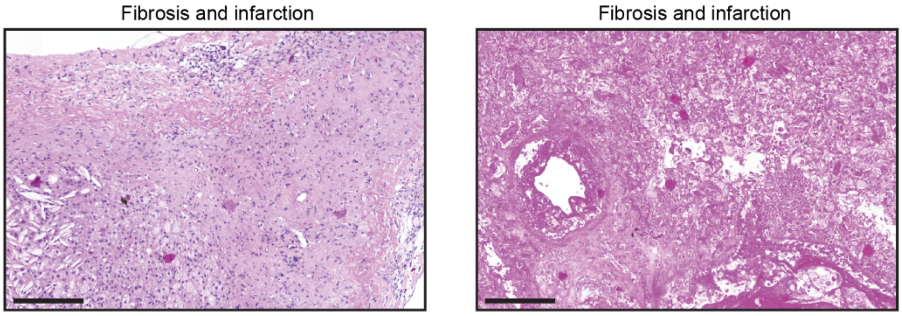

Supplementary Fig. 10. Representative histological examples illustrating scoring. An adapted International Society for Heart and Lung Transplantation (ISHLT) scoring was used to quantify allograft rejection, including ISHLT grade A (acute rejection), grade B (airway inflammation), and grade C (chronic airway rejection) scoring. (**a**) Using ISHLT grade A, acute rejection was scored as follows. A0, no evidence of acute rejection. A1, presence of sparse lymphocyte layers around blood vessels that are not visible on low magnification. A2, prominent perivascular lymphocytic infiltrates that are easily recognizable on low magnification. A3, extension of perivascular lymphocytic infiltrates into adjacent alveolar septa. A4, diffuse perivascular, interstitial and alveolar inflammation and necrotizing vasculitis. (**b**) ISHLT grade B differentiated the following features of airway inflammation. B0, no evidence of airway inflammation. B1, lymphocytes in walls of small airways without epithelial damage. B2, significant numbers of lymphocytes in walls of small airways, extending into bronchiolar walls, intraepithelial lymphocytosis, and ulceration of airway walls. (**c**) ISHLT grade C served to quantify chronic airway rejection. C0, no evidence of chronic airway rejection. C1, connective tissue completely obliterates the lumen of an airway. (**d**) Exemplary hematoxylin and eosin staining showing allografted lung tissue with advanced fibrosis and infarction (abbreviated as F/I in scores) due to allograft rejection. Black arrows indicate blood vessels and blue arrows indicate airways. Scale bars = 200 µm. Representative images of n ≥ 6 mice of 2–3 independent experiments.

Supplementary Table 1. Antibodies used for flow cytometry.

Target	Fluorophore	Clone	Dilution	Provider, catalog number
CD3e	PerCP-Cy5.5	145-2C11	1:200	Biolegend, #100218
CD3e	BV605	145-2C11	1:200	Biolegend, #100237
CD4	V450	GK1.5	1:400	Thermo Fisher, #560468
CD4	PE-Dazzle-594	GK1.5	1:600	Biolegend, #100565
CD8a	BUV395	53-6.7	1:200	BD Biosciences, #563786
CD11b	BV785	M1/70	1:600	Biolegend, #101243
CD19	BV510	6D5	1:400	Biolegend, #115546
CD19	PE-Cy7	6D5	1:400	Biolegend, #115509
CD25	APC	PC61	1:200	Biolegend, #102012
CD25	BV650	PC61	1:400	Biolegend, #102038
CD45.2	FITC	104	1:200	Biolegend, #109806
CD45.2	AF700	104	1:400	Biolegend, #109822
CD90.2	BUV737	53-2.1	1:400	BD Biosciences, # 741701
CD122	APC	TMB1	1:200	Biolegend, #123214
Foxp3	PE	FJK-16s	1:200	Thermo Fisher, #12-5773-82
Foxp3	AF488	FJK-16s	1:200	Thermo Fisher, #53-5773-82
Ki-67	BV421	B56	1:200	BD Biosciences, #562899
NK1.1	AF700	PK136	1:200	Biolegend, #108730
NK1.1	BV711	PK136	1:200	Biolegend, #108745
Live/Dead	eFlour 780		1:1000	Thermo Fisher, # 65-0865-14

Supplementary Table 2. Antibodies used for immunofluorescence staining.

Target	Fluorophore	Clone	Dilution	Provider, catalog number
B220	Alexa 488	RA3-6B2	1:100	Biolegend, #103225
CD3e	Alexa 647	145-2C11	1:100	Biolegend, #100322
CD4	Alexa 594	GK1.5	1:100	Biolegend, #100446
CD8a	Alexa 488	53-6.7	1:100	Biolegend, #100723
CD11c	Alexa 594	N418	1:200	Biolegend, #117346
Foxp3	eFluor 570	FJK-16s	1:100	Thermo Fisher, #41-5773-82
PNAd	Alexa 647	MECA-79	1:200	Biolegend, #120808
SMA	eFlour660	1A4	1:300	Thermo Fisher, 50-9760-82
