# Supplementary material for: Biased IL-2 signals induce Foxp3-rich pulmonary lymphoid structures and facilitate long-term lung allograft acceptance in mice
Source: Nat Commun. 2023 Mar 13;14:1383. doi: 10.1038/s41467-023-36924-z (PMC10011523; doi:10.1038/s41467-023-36924-z)
Supplement: Supplementary file 2 — Reporting Summary [file 41467_2023_36924_MOESM2_ESM.pdf]

nature portfolio

Corresponding author(s): Onur Boyman

Last updated by author(s): Jan 23, 2023

Reporting Summary

Nature Portfolio wishes to improve the reproducibility of the work that we publish. This form provides structure for consistency and transparency in reporting. For further information on Nature Portfolio policies, see our Editorial Policies and the Editorial Policy Checklist.

Statistics

For all statistical analyses, confirm that the following items are present in the figure legend, table legend, main text, or Methods section.

n/a	Confirmed	
☐	☒	The exact sample size (*n*) for each experimental group/condition, given as a discrete number and unit of measurement
☐	☒	A statement on whether measurements were taken from distinct samples or whether the same sample was measured repeatedly
☐	☒	The statistical test(s) used AND whether they are one- or two-sided<br>*Only common tests should be described solely by name; describe more complex techniques in the Methods section.*
☐	☒	A description of all covariates tested
☐	☒	A description of any assumptions or corrections, such as tests of normality and adjustment for multiple comparisons
☐	☒	A full description of the statistical parameters including central tendency (e.g. means) or other basic estimates (e.g. regression coefficient) AND variation (e.g. standard deviation) or associated estimates of uncertainty (e.g. confidence intervals)
☐	☒	For null hypothesis testing, the test statistic (e.g. *F*, *t*, *r*) with confidence intervals, effect sizes, degrees of freedom and *P* value noted<br>*Give P values as exact values whenever suitable.*
☒	☐	For Bayesian analysis, information on the choice of priors and Markov chain Monte Carlo settings
☒	☐	For hierarchical and complex designs, identification of the appropriate level for tests and full reporting of outcomes
☒	☐	Estimates of effect sizes (e.g. Cohen's *d*, Pearson's *r*), indicating how they were calculated

Our web collection on statistics for biologists contains articles on many of the points above.

Software and code

Policy information about availability of computer code

Data collection	Flow Cytometry: BD FACSDiva 8.0<br>Confocal Image: Leica LAS-X v3.7
Data analysis	Flow Cytometry: FlowJo v10<br>Statistics: GraphPad Prism v8<br>Image Analysis: Imaris v9.3, Microscopy Image Browser (MIB) v2.6<br>RStudio 1.4.1103

For manuscripts utilizing custom algorithms or software that are central to the research but not yet described in published literature, software must be made available to editors and reviewers. We strongly encourage code deposition in a community repository (e.g. GitHub). See the Nature Portfolio guidelines for submitting code & software for further information.

Data

Policy information about availability of data

All manuscripts must include a data availability statement. This statement should provide the following information, where applicable:

- Accession codes, unique identifiers, or web links for publicly available datasets
- A description of any restrictions on data availability
- For clinical datasets or third party data, please ensure that the statement adheres to our policy

All data and code are available in the main text, methods or the extended data.

Human research participants

Policy information about studies involving human research participants and Sex and Gender in Research.

Reporting on sex and gender	Not applicable
Population characteristics	Not applicable
Recruitment	Not applicable
Ethics oversight	Not applicable

Note that full information on the approval of the study protocol must also be provided in the manuscript.

Field-specific reporting

Please select the one below that is the best fit for your research. If you are not sure, read the appropriate sections before making your selection.

[x] Life sciences [ ] Behavioural & social sciences [ ] Ecological, evolutionary & environmental sciences

For a reference copy of the document with all sections, see nature.com/documents/nr-reporting-summary-flat.pdf

Life sciences study design

All studies must disclose on these points even when the disclosure is negative.

Sample size	The total number of transplantations performed for histological analyses were 105, including on day 5 (PBS: n = 15, IL-2cx: n = 18), day 15 (PBS: n = 10, IL-2cx: n = 10), day 30 (PBS: n = 9, IL-2cx: n = 13), day 60 (PBS: n = 8, IL-2cx: n = 9), and day 90 (PBS: n = 6, IL-2cx: n = 7). These numbers do not include additional transplantations done for the measurements of PaO2 (n = 16) and the application of diphtheria toxin (DT) in Foxp3DTR (n = 11).<br>Sample size was calculated using power.anova.test to detected an incidence difference (tolerance) of 60% with alpha 0.05, power = 0.8, which results in a sample size of n = 8.
Data exclusions	No data were excluded.
Replication	The number of experimental replicates is indicated in the figure legends.
Randomization	Mice were randomly allocated to individual treatment groups.
Blinding	Where possible, investigators were blinded.<br>Fig. 1, d-f: Scoring of rejection was performed by blinded investigator.<br>Fig. 2, b-c: Blinding was not possible, as measurement of PaO2 and compliance requires surgical skill of investigator.<br>FIg. 3: Scoring of rejection was performed by blinded investigator.<br>Fig. 4: Data analysis was performed by blinded investigator.<br>Figs. 5 and 6: Staining and image aquisition was performed by blinded investigator.<br>Fig. 7, a: Staining and image aquisition was performed by blinded investigator; b: data analysis was performed by blinded investigator.

Reporting for specific materials, systems and methods

We require information from authors about some types of materials, experimental systems and methods used in many studies. Here, indicate whether each material, system or method listed is relevant to your study. If you are not sure if a list item applies to your research, read the appropriate section before selecting a response.

Materials & experimental systems

n/a	Involved in the study
	☒ Antibodies
☒	☐ Eukaryotic cell lines
☒	☐ Palaeontology and archaeology
	☒ Animals and other organisms
☒	☐ Clinical data
☒	☐ Dual use research of concern

Methods

n/a	Involved in the study
☒	☐ ChIP-seq
	☒ Flow cytometry
☒	☐ MRI-based neuroimaging

Antibodies

Antibodies used

Antibodies used for flow cytometry (target, fluorophore, clone, dilution, provider, art nr):
CD3e,PerCP-Cy5.5,145-2C11,1:200,Biolegend, #100218
CD3e,BV605,145-2C11,1:200,Biolegend, #100237
CD4,V450,GK1.5,1:400,Thermo Fisher, #560468
CD4,PE-Dazzle-594,GK1.5,1:600,Biolegend, #100565
CD8a,BUV395 53-6.7,1:200,BD Biosciences, #563786
CD11b,BV785,M1/70,1:600,Biolegend, #101243
CD19,BV510,6D5,1:400,Biolegend, #115546
CD19,PE-Cy7,6D5,1:400,Biolegend, #115509
CD25,APC,PC61,1:200,Biolegend #102012
CD25,BV650,PC61,1:400,Biolegend, #102038
CD45.2,FITC,104,1:200,Biolegend, #109806
CD45.2,AF700,104,1:400,Biolegend, #109822
CD90.2,BUV737,53-2.1,1:400,BD Biosciences, # 741701
CD122,APC,TMB1,1:200,Biolegend, #123214
Foxp3,PE,FJK-16s,1:200,Thermo Fisher, #12-5773-82
Foxp3,AF488,FJK-16s,1:200,Thermo Fisher, #53-5773-82
Ki-67,BV421,B56,1:200,BD Biosciences, #562899
NK1.1,AF700,PK136,1:200,Biolegend, #108730
NK1.1,BV711,PK136,1:200,Biolegend, #108745
Live/Dead,eFlour 780,-,1:1000,Thermo Fisher, # 65-0865-14

Antibodies used for immunofluorescence staining (target, fluorophore, clone, dilution, provider, art nr)::
B220,Alexa 488,RA3-6B2,1:100,Biolegend, #103225
CD3e,Alexa 647,145-2C11,1:100,Biolegend, #100322
CD4,Alexa 594,GK1.5,1:100,Biolegend, #100446
CD8a,Alexa 488,53-6.7,1:100,Biolegend, #100723
CD11c,Alexa 594 N418,1:200,Biolegend, #117346
Foxp3,eFluor 570,FJK-16s,1:100,Thermo Fisher, #41-5773-82
PNAd,Alexa 647,MECA-79,1:200,Biolegend, #120808
SMA,eFlour660,1A4,1:300,Thermo Fisher, #50-9760-82

Antibodies used for IHC:
CD3 (IR503, DAKO)
anti-mouse CD4 (14-9766, eBioscience)
anti-mouse CD8 (14-0808, eBioscience)
anti-rat B220 (550539, BD Pharmingen)
anti-rabbit FoxP3 (ab54501, Abcam)

anti-rat IgG/HRP (A 5795, Sigma-Aldrich)
anti-goat/HRP (P0160, DAKO)
and anti-rabbit/HRP (K4003, DAKO)

Validation

Antibodies were validated by respective manufacturers for flow cytometry by staining mouse primary cells or cell lines. Validation statements as well as references from the literature can be found on the manufacturers' websites. Antibodies were not validated by/for flow cytometry, immunofluorescence staining or IHC.
Immunofluorescence and IHC antibodies were re-validated internally on spleen as positive control and non-hematopoetic tissue as negative control.

Animals and other research organisms

Policy information about [studies involving animals](); [ARRIVE guidelines]() recommended for reporting animal research, and [Sex and Gender in Research]()

Laboratory animals	3-month-old female C57BL/6J mice, mice expressing diphtheria toxin (DT) receptor under the Foxp3 promoter (Foxp3DTR; B6 background; The Jackson Laboratory), and BALB/c (Charles River) mice were purchased from indicated suppliers.
Wild animals	No wild animals were used in the study.
Reporting on sex	Animals used were females or males.
Field-collected samples	No field collected samples were used in the study.
Ethics oversight	Animal experiments received prior approval by the veterinary office of Canton of Zurich (license number ZH240/15), following pre-established exclusion criteria, and were conducted in accordance with Swiss Federal and Cantonal laws. Certain revision experiments were conducted at Kyoto University (institutional license number Med Kyo 22569) in accordance with Japanese laws.

Note that full information on the approval of the study protocol must also be provided in the manuscript.

Flow Cytometry

Plots

Confirm that:

- [x] The axis labels state the marker and fluorochrome used (e.g. CD4-FITC).
- [x] The axis scales are clearly visible. Include numbers along axes only for bottom left plot of group (a 'group' is an analysis of identical markers).
- [x] All plots are contour plots with outliers or pseudocolor plots.
- [x] A numerical value for number of cells or percentage (with statistics) is provided.

Methodology

Sample preparation	Single cell suspensions of lungs, LNs, spleen, and blood were prepared following published protocols (refs. 58-61). In brief, cells were stained for flow cytometry analysis using PBS containing 1% FCS and 2 mM EDTA with fluorochrome-conjugated antibodies. Antibodies are listed in Supplementary Table 1. For Foxp3 staining, cells were fixed and permeabilized with a staining kit (eBioscience) according to manufacturer's instructions.
Instrument	Data were acquired using BD LSR II Fortessa (BD Biosciences).
Software	Data were analyzed by using FlowJo (BD Biosciences).
Cell population abundance	Cell sorting was not used in this study.
Gating strategy	The markers used to define immune cell population are provided in the figures. Cells: FSC/SSC gate was set to include all leukocytes but exclude cell debris. FSC-H/FSC-A gate was used to gate on singlets. Dead cells were excluded by Fixable Viability Dye eFlour780. Lymphocytes were gated on CD45.2+ cells. CD19 was used to separate B cells and non-B cells. CD90.2 was used to gate T cells from CD19- cells. NK1.1 and CD122 were used to distinguish NKT cells from T cells. Following populations were gated from T (non-NKT) cells: CD4+CD8- (CD4) and CD4-CD8+(CD8) T cells. Within CD4 T cells, Foxp3 was used to gate on Foxp3+ Treg cells. From the CD45+CD19-CD90.2- population the following populations were gated: NK1.1 +CD122+ (NK cells) and NK1.1-CD122-CD11b+ (myeloid) cells.

- [x] Tick this box to confirm that a figure exemplifying the gating strategy is provided in the Supplementary Information.
